# Associations of accelerometer measured school- and non-school based physical activity and sedentary time with body mass index: IPEN Adolescent study

**DOI:** 10.1186/s12966-022-01324-x

**Published:** 2022-07-14

**Authors:** Delfien Van Dyck, Anthony Barnett, Ester Cerin, Terry L. Conway, Irene Esteban-Cornejo, Erica Hinckson, Lukáš Rubín, Elaine Rush, Orna Baron-Epel, Kelli L. Cain, Lars Breum Christiansen, Mohammed Zakiul Islam, Josef Mitáš, Javier Molina-García, Adewale Oyeyemi, Harish Ranjani, Rodrigo Reis, Maria Paula Santos, Cindy Sit, Anna Timperio, Wan Abdul Manan Wan Muda, James F. Sallis

**Affiliations:** 1 Faculty of Medicine and Health Sciences, Department of Movement and Sports Sciences, Ghent University, Ghent, Belgium; 2 Mary MacKillop Institute for Health Research, Australian Catholic University, Melbourne, Australia; 3 School of Public Health, The University of Hong Kong, Hong Kong, China; 4 Baker Health and Diabetes Institute, Melbourne, Australia; 5 Herbert Wertheim School of Public Health and Human Longevity Science, University of California San Diego, La Jolla, San Diego, CA USA; 6 PROmoting FITness and Health Through Physical Activity Research Group, Department of Physical Education and Sports, Faculty of Sport Sciences, Sport and Health University Research Institute (iMUDS), University of Granada, Granada, Spain; 7 Faculty of Health and Environmental Science, School of Sport and Recreation, Auckland University of Technology, Auckland, New Zealand; 8 Institute of Active Lifestyle, Faculty of Physical Culture, Palacký University Olomouc, Olomouc, Czech Republic; 9 Department of Physical Education and Sport, Faculty of Science, Humanities and Education, Technical University of Liberec, Liberec, Czech Republic; 10 University of Haifa, Haifa, Israel; 11 Department of Sports Science and Clinical Biomechanics, University of Southern Denmark, Odense, Denmark; 12 Department of Architecture, Bangladesh University of Engineering & Technology (BUET), Dhaka, Bangladesh; 13 AFIPS Research Group, Department of Musical, Visual and Corporal Expression Teaching, University of Valencia, Valencia, Spain; 14 Department of Physiotherapy, College of Medical Sciences, University of Maiduguri, Maiduguri, Nigeria; 15 Madras Diabetes Research Foundation, Chennai, India; 16 Prevention Research Center, Brown School, Washington University, St Louis, USA; 17 Graduate Program in Urban Management, Pontifical Catholic University of Parana, Curitiba, Brazil; 18 Faculty of Sport, Research Centre in Physical Activity, Health and Leisure (CIAFEL), University of Porto, Porto, Portugal; 19 Department of Sports Science and Physical Education, The Chinese University of Hong Kong, Hong Kong, China; 20 Institute for Physical Activity and Nutrition (IPAN), School of Exercise and Nutrition Sciences, Deakin University, Geelong, Australia; 21 Graduate School of Public Health, Alma Ata University, Yogyakarta, Indonesia

**Keywords:** Adolescents, Exercise, Body weight, Physical activity, Public health

## Abstract

**Background:**

This study examined the strength, shape and direction of associations of accelerometer-assessed overall, school- and non-school-based moderate-to-vigorous physical activity (MVPA) and sedentary time (ST) with BMI among adolescents across the world. Second, we examined whether these associations differed by study site and sex.

**Methods:**

Cross-sectional data from the IPEN Adolescent study, an observational multi-country study, were used. Participants wore an accelerometer for seven days, reported height and weight, and completed a socio-demographic survey. In total, 4852 adolescents (46.6% boys), aged 11–19 years (mean age = 14.6, SD = 1.7 years) were included in the analyses, using generalized additive mixed models.

**Results:**

Adolescents accumulated on average 41.3 (SD = 22.6) min/day of MVPA and 531.8 (SD = 81.1) min/day of ST, and the prevalence of overweight and obesity was 17.2% (IOTF), but these mean values differed by country. Linear negative associations of accelerometer-based MVPA and ST with standardized BMI scores and the likelihood of being overweight/obese were found. School-based ST and non-school-based MVPA were more strongly negatively associated to the outcomes than non-school based ST and school-based MVPA. Study site moderated the associations; adolescent sex did not. No curvilinear associations were found.

**Conclusions:**

This multi-country study confirmed the importance of MVPA as a potential protective factor against overweight/obesity in adolescents. Non-school-based MVPA seemed to be the main driver of these associations. Unexpected results were found for ST, calling for further examination in methodologically sound international studies but using inclinometers or pressure sensors to provide more precise ST measures.

**Supplementary Information:**

The online version contains supplementary material available at 10.1186/s12966-022-01324-x.

## Background

Worldwide, levels of overweight and obesity in adolescents are alarmingly high. Since 2000, the increasing trends in mean body mass index (BMI) in adolescents have plateaued in high-income countries, but this plateau occurred at high prevalence. However, in East-, South- and Southeast Asia, mean BMI levels are still rapidly increasing [1]. Overweight or obesity in adolescence is associated with many negative physical, psychosocial and mental health consequences throughout the life-course [2–5]. Consequently, variables associated with a healthy weight in adolescents should be identified to inform prevention strategies.

Increased physical activity (PA) and reduced sedentary time (ST) are important cornerstones in the prevention and management of overweight and obesity [6]. A large-scale observational study using International Children physical Activity Database (ICAD) accelerometer data showed greater duration and intensity of PA were consistently associated with lower BMI in children and adolescents [7]. Crowe et al. [8] found higher PA may be more critical than lower screen time in the association with overweight and obesity in adolescents. Recently published results from the UK ALSPAC study showed that total accelerometer-assessed sedentary time was not, but higher TV viewing time was prospectively associated with a higher fat mass index in both active and inactive adolescents [9]. A recent meta-analysis concluded the majority of evidence supports the hypothesis that ST has no prospective association with adiposity in youth, while prospective associations between MVPA and adiposity were inconsistent [10]. Overall, current data suggest MVPA may be more important than ST in relation to weight-related outcomes in youth.

Although previous observational single- and multi-country studies examined how total levels of PA and ST relate to BMI in adolescents, some gaps remain. First, the shape of associations is unclear. All previous studies only examined linear associations, but some associations might be curvilinear, meaning relations may attenuate or strengthen at higher or lower levels of PA/ST. In a multi-country adults study, a curvilinear relation of accelerometer-based MVPA with BMI has been reported: associations were negative, but weakened at higher levels of MVPA [11]. Such a potential curvilinearity has not been examined yet in adolescents [12]. Second, available international databases (e.g. ICAD) mainly include accelerometer data from European, Australian and North American adolescents [7, 10]. Data from other continents (e.g., Asia and Africa) are lacking, and associations might differ there. Third, although studies examining patterns of accumulation (e.g. prolonged bouts and breaks) are emerging [7], the predominant focus of the existing studies has been on the total volume of MVPA or ST. Instead of only examining associations of total MVPA or ST with BMI, examining associations of MVPA and ST accumulated in different settings (e.g. at school or outside of school) with BMI may help to inform the development of setting-specific interventions to address overweight and obesity.

Based on these gaps in literature, our first aim was to examine the strength, shape, and direction of the associations of accelerometer-assessed overall, school- and non-school-based MVPA and ST with standardized BMI and overweight/obesity status in 15 countries. The second aim was to establish whether these associations differed according to study site and sex.

## Methods

### Study design

The IPEN Adolescent study was an observational, multi-country, cross-sectional study with purposive sampling, including 18 cities/regions (hereafter, sites) in 15 geographically, economically and culturally diverse countries across six continents: Australia (AUS; Melbourne), Bangladesh (BGD; Dhaka), Belgium (BEL; Ghent), Brazil (BRA; Curitiba), Czech Republic (CZE; Olomouc, Hradec Králové), Denmark (DNK; Odense), Hong Kong SAR (CHN; Hong Kong), India (IND; Chennai), Israel (ISR; Haifa), Malaysia (MYS; Kuala Lumpur), New Zealand (NZL; Auckland, Wellington), Nigeria (NGA; Gombe), Portugal (PRT; Porto region), Spain (ESP; Valencia) and USA (Baltimore and Seattle regions). Data were collected between 2009 and 2016. Detailed information regarding sites, protocol, design, and measures is presented in a protocol paper [13]. In short, recruited adolescent participants were between 11 and 19 years, along with one parent or legal guardian (exception New Zealand: only adolescents), who were living in neighbourhoods (i.e., compilation of administrative units) chosen to maximize variance in neighbourhood income and walkability. For categorisation on walkability, a Geographic Information Systems (GIS)-based walkability index was used that was a composite of residential density, intersection density and land use mix [14, 15]. Malaysia, Nigeria, and India relied on local knowledge to identify diverse neighbourhoods. In most countries, the socio-economic status (SES) of neighbourhoods was classified as low or high based on city/region-specific demographic data. Neighbourhoods were stratified into high walkability-high SES, high walkability-low SES, low walkability-high SES and low walkability-low SES quadrants for balancing representation of neighbourhood types during participant recruitment.

### Participant recruitment

Two strategies were used: (1) systematic selection of potential participants living at an address within the preselected neighbourhoods (Brazil, Israel, USA), and (2) recruiting participants from preselected schools located within the four quadrants (10 countries). Belgium and India combined both strategies. Recruitment within schools was conducted using random sampling, classroom or year-level recruitment. After recruitment, the adolescents’ residential address was assigned to the appropriate quadrant code of the neighbourhood where they lived. All countries conducted recruitment in person, except in the USA telephone and mail methods were used. In total, 6950 adolescents participated in the study. Mean participation rate, based on the countries that provided this information, was 48.4% (SD 23.6%). The lowest participation rate was reported in India (11%) and New Zealand (12.8%); participation was highest in Czech Republic (89.7%). Additional information like recruitment dates, site-specific participation rates, school schedules, contact mode and incentives in each country are reported elsewhere [13]. All studies in each country were approved by their Institution’s Ethics Committees, and participants and their legal guardian provided informed assent/consent.

### Measures

#### BMI

Weight and height were self-reported in eight countries, and measured by research assistants in seven countries [13]. Sex was self-reported and decimal age in years calculated from birth date to date of measurement. To have wider international representation of sex- and age-adjusted BMI standards, the LMS Growth software program was used [16], applying the 2007 WHO Child Growth Reference [17] and the International Obesity Task Force (IOTF) cut points [18, 19]. The program converts physical assessments to age- and sex-adjusted BMI standard deviation (SD) scores (based on the 2007 WHO Child Growth reference) and IOTF grades. The IOTF cut-offs classify BMI in children aged 2–18 years as thin (3 grades), normal weight, overweight, or obese. The six possible IOTF grades reflect the adjusted BMI values projected to adult BMI cut-offs at age 18: thinness grade -3 (BMI < 16), thinness grade -2 (BMI 16 to < 17), thinness grade -1 (BMI 17 to < 18.5), normal weight grade 0 (BMI 18.5 to < 25), overweight grade + 1 (BMI 25 to < 30) or obese grade + 2 (BMI 30 +). For this study, IOTF grades were reclassified into thin/normal versus overweight/obese. Finally, the Centers for Disease Control (CDC) BMI-SD scores [20] were also considered in sensitivity analyses to examine whether using WHO BMI-SD scores versus CDC BMI-SD scores produced different results (see Appendices 1, 2, 3 and 4).

#### Accelerometer-assessed MVPA and ST

Adolescents (all or a subsample, depending on study site; *n* = 5215) were asked to wear an ActiGraph accelerometer on the right hip for at least seven days during waking hours when not swimming or bathing. Due to varying availability across study sites, four ActiGraph models were used (7164, GT1M, GT3X and GT3X +). To standardize screening and scoring procedures, accelerometer data from all countries were sent to the study’s Coordinating Center site. Trained researchers at the coordinating center screened all data using MeterPlus v.5.0 to ensure comparable data processing and scoring methods across all sites. Screening procedures were checked for devices that had malfunctioned, flagged non-wearing time for exclusion, and marked valid wearing days for scoring. More details about IPEN Adolescent accelerometer scoring protocols can be found on the IPEN website at http://ipenproject.org/methods_accelerometers.html.

All accelerometer vertical axis data were collected with (or converted to) a 30-s epoch, which was the shortest length that could be standardized across all study sites. While a 60-s epoch has often been used in both adult and youth studies, shorter epochs appear to record more accurately the intermittent, short bursts of physical activity common in young people [21]. Non-wear time was defined as 60 + minutes of consecutive zero counts, which is an interval that very accurately differentiates sedentary behavior from non-wear time in adolescents [22]. A valid wearing day consisted of at least 8 h of wear time during waking hours from 6AM to midnight. Only participants with at least 4 valid wearing days were included in the analyses (n = 4852). The wearing criteria of at least 8 h per day to define a valid day and at least 4 valid wearing days for inclusion in analyses are commonly used in adolescent accelerometer studies [21].

Evenson cut-points for MVPA and ST (≤ 100 counts per minute) were applied to compute average duration per day (minutes/day) across all valid wearing days [23]. In addition, MVPA and ST durations ‘during school’ on school days and during all ‘non-school’ periods (i.e., before and after school on school days plus all valid wearing hours on non-school days) were extracted. Self-reported school start and end times were used in most countries to determine school days and in-school times. These data were not available in the USA and 08:15 AM to 02:15 PM was used as an estimate of the school day on weekdays for the USA [13].

Fourteen countries used an Actigraph GT model (GT1M, GT3X, or GT3X +), and one country (USA) primarily used the older generation 7164 model. For sites using GT models, protocols specified that the Low Frequency Extension (LFE) be enabled because it produces comparability between data collected with the older 7164 model and the newer generation GT models [24]. Twelve countries using the GT models always had the LFE enabled when collecting accelerometer data (total of 4482 cases). However, two countries had some wearings that used a GT model with the normal filter enabled, which made it less sensitive to lower-intensity activity (90 cases in the USA and 154 cases in India). One country (Denmark) used the normal filter for all accelerometer wearings (126 cases). To account for potential effects of using less sensitive GT models with the normal filter enabled [23], a variable denoting comparability of accelerometer models was created (0 = non-comparable; 1 = comparable) and used in sensitivity analyses. The 7164 and GT models with LFE were considered comparable (*n* = 4482 cases); GT models with the normal filter used (*n* = 370) were considered non-comparable to the 7164 and GT models with LFE enabled [24].

#### Socio-demographic covariates and study design measures

Sex, age and highest educational attainment in the household were included as covariates in all statistical models. Study design variables adjusted for included site (city/region) and the dichotomous (low versus high) indicators of within-site administrative-unit walkability and SES. To adjust for accelerometer-related differences across participants, number of valid days of accelerometer wear time, average accelerometer wear time/day, and accelerometer comparability (yes vs. no) were included in analyses. Recruitment-related clustering within residential census units and school attended was adjusted for by including administrative codes for neighbourhoods and schools as random effects in analyses.

### Data Analyses

Descriptive statistics were computed for all relevant variables, by site and for the whole sample. Missing data for at least one variable occurred in 9% of participants, with a minimum of 0% in Hong Kong (CHN) and maximum of 56.7% in Melbourne (AUS). The presence of missing data on specific variables was related to other study variables, i.e., data were at least missing at random (MAR) rather than completely missing at random [25]. Specifically, in the analytical sample (*n* = 4852; adolescents with valid accelerometer data), six variables were associated with having missing values on one or more variables examined in this study. Older adolescents (OR = 1.167; 95%CI: 1.012, 1.236; *p* < 0.001), females (OR = 1.258; 95%CI: 1.037, 1.526; *p* = 0.020) and those with more MVPA minutes per valid day (OR = 1.011; 95%CI: 1.007, 1.015; *p* < 0.001) were more likely to have missing values. Adolescents with more valid days of accelerometer wear (OR = 0.827; 95%CI: 0.768, 0.892; *p* < 0.001), more total wear time per valid day (OR = 0.997; 95%CI: 0.996, 0.998; p < 0.001) and more ST per day (OR = 0.998 95%CI: 0.996, 0.999; *p* = 0.001) were less likely to have missing values. As analyses based on complete data only when missing data are MAR can yield biased results, while analyses based on properly-conducted multiple imputations do not [25], ten imputed datasets were created for the main regression analyses [26]. We also conducted the same analyses on cases with complete data (*n* = 4384) for sensitivity analysis purposes. Multiple imputations were performed using chained equations (MICE) accounting for within-city administrative-unit- and school-level clustering effects arising from the two-stage stratified sampling strategy employed in each study site [26]. The ten imputed datasets were created in R (R Development Core Team, 2020) using the package ‘mice’ and following procedures outlined by van Buuren [26].

For the first aim, we used generalized additive mixed models (GAMMs) with random intercepts at the within-city administrative-unit and school level [11, 27]. As BMI variables were continuous and approximately normally distributed, they were modelled using GAMMs with Gaussian variance and identity link functions. As BMI status (dichotomized IOTF grades) was a binary variable it was modelled using GAMMs with binomial variance and logit link functions. The reported antilogarithms of the regression coefficient estimates of the binomial GAMMs represent odds ratios of inclusion in the overweight/obese IOTF category.

Main-effect sets of GAMMs estimated relationships of total MVPA and ST (Model 1), as well as school-based MVPA and ST and non-school-based MVPA and ST (Model 2), with the outcome variables, adjusting for adolescent sex, age, site, highest education level, within-city/region administrative-unit-level walkability and SES, accelerometer comparability, number of valid days of accelerometer wear time and average accelerometer wear time per day. There was no collinearity between the explanatory variables included in the GAMMs (maximum absolute correlation = 0.30). Curvilinear relationships of MVPA and ST variables with BMI outcomes were estimated using non-parametric smooth terms in GAMMs, which were modelled using thin-plate splines [27]. Smooth terms failing to provide evidence of a curvilinear relationship (an Akaike’s Information Criterion [AIC] value 10 + units smaller than the linear model) were replaced by simpler linear terms.

Separate GAMMs were run to estimate MVPA and ST variables by sex, site (second study aim) and accelerometer comparability (sensitivity analyses) interaction effects on BMI outcomes. The significance of interaction effects of site (each consisting of 17 PA or ST variable-by-site interaction terms) was evaluated by comparing AIC values of models with and without interaction effects, whereby the model with a ≥ 10-unit smaller AIC was preferred [28]. The significance of the interaction effects of sex and accelerometer comparability (defined by a single interaction term) were determined using the Wald test. Significant interaction effects were probed by computing the associations of MVPA and ST variables with BMI outcomes at different values of the moderator. Sensitivity analyses for the WHO BMI-SD score as outcome were undertaken by running the same GAMMs with the CDC BMI-SD scores as outcome (Appendices 1, 2, 3 and 4). All analyses were conducted on the imputed data sets (primary analyses reported in the manuscript) and cases with complete data (sensitivity analyses, Appendices 1, 2, 3 and 4). All analyses were conducted in R (R Development Core Team, 2020) using the packages ‘car’ [29], ‘mgcv’ [27], ‘gmodels’ [30], and ‘mice’ [31].

## Results

### Descriptive characteristics

On average, adolescents accumulated 41.3 (SD = 22.6) min/day of MVPA and 531.8 (SD = 81.1) min/day of ST. Average total MVPA (mins/day) was higher in the Czech, New Zealand and Portuguese study sites and in Gombe (NGA), and lower in Chennai (IND) and Kuala Lumpur (MYS). Average total ST was substantially lower in Olomouc (CZE) and higher in Valencia (ESP). Approximately 15% of the sample was classified as being overweight and 5.0% as being obese. The prevalence of overweight and obesity ranged from 7.5% in Gombe (NGA) and 7.9% in Odense (DNK) to 31.9% in Curitiba (BRA) and 32.2% in the Porto region (PRT) (Table 1).

**Table 1:** Descriptive statistics for the whole sample and by study site

Countries	All	AUS	BGD	BEL	BRA	CZE		DNK	HKG	IND	ISR	MYS	NZL		NGA	PRT	ESP	USA	
Cities	All sites	Melbourne	Dhaka	Ghent	Curitiba	Olomouc	Hradec Králové	Odense	Hong Kong	Chennai	Haifa	Kuala Lumpar	Auckland	Wellington	Gombe	Porto region	Valencia	Baltimore	Seattle
Overall N	4852	372	90	224	419	56	49	126	549	315	223	325	340	160	245	143	373	438	405
**Age (years)**																			
Mean	14.6	14.9	13.9	13.3	14.1	13.9	15.8	13.0	14.4	13.8	15.3	14.6	14.8	14.5	15.3	15.9	16.6	14.1	14.0
Std Dev	1.7	1.6	1.8	1.4	1.7	2.0	1.7	1.2	1.7	1.5	1.4	1.2	1.4	1.3	1.6	1.2	0.8	1.4	1.4
**Gender**																			
Male	46.6%	38.7%	53.3%	39.7%	48.7%	33.9%	49.0%	38.1%	45.4%	52.4%	39.9%	40.3%	42.1%	88.4%	51.1%	37.1%	43.4%	46.6%	53.6%
**Highest education in the household**																			
< College degree	39.6%	18.5%	42.2%	22.8%	57.3%	30.4%	4.1%	28.6%	63.2%	52.4%	37.2%	50.5%	38.2%	21.9%	42.9%	48.3%	44.0%	25.1%	23.2%
≥ College degree	53.1%	35.5%	57.8%	75.4%	42.7%	28.6%	40.8%	70.6%	36.8%	47.3%	61.4%	35.4%	50.9%	71.3%	54.3%	36.4%	56.0%	74.2%	76.8%
Missing	7.3%	46.0%	0%	1.8%	0%	41.1%	55.1%	0.8%	0%	0.3%	1.3%	14.2%	10.9%	6.9%	2.9%	15.4%	0%	0.7%	0%
**Child IOTF grade for adjusted BMI**																			
Thinness 3 (BMI < 16)	1.9%	0.5%	3.3%	1.3%	1.2%	0%	0%	0%	0.4%	5.7%	0.9%	2.8%	0.3%	0%	15.1%	0%	0.5%	1.1%	0.2%
Thinness 2 (BMI 16 to < 17)	3.0%	1.3%	2.2%	3.1%	1.2%	1.8%	0%	3.2%	2.4%	9.2%	2.7%	5.8%	0.3%	0%	15.1%	0%	0.8%	1.4%	0.5%
Thinness 1 (BMI 17 to < 18.5)	7.6%	4.6%	14.4%	9.4%	4.5%	14.3%	2.0%	7.1%	11.1%	14.9%	6.3%	10.5%	4.7%	3.8%	14.3%	2.8%	6.4%	4.6%	4.4%
Normal weight 0 (BMI 18.5 to < 25)	64.5%	54.3%	54.4%	66.1%	61.1%	73.2%	79.6%	77.8%	76.0%	45.4%	74.0%	54.8%	70.6%%	74.4%	47.8%	54.5%	70.8%	65.5%	70.9%
Overweight 1 (BMI 25 to < 30)	15.2%	14.5%	14.4%	7.6%	23.0%	8.9%	16.3%	7.9%	8.4%	17.8%	11.7%	15.1%	17.4%	14.4%	6.9%	25.9%	17.7%	19.9%	16.8%
Obese 2 (BMI 30 +)	5.0%	4.3%	4.4%	1.3%	8.9%	1.8%	2.0%	0%	1.8%	7.0%	2.7%	8.3%	5.9%	6.3%	0.6%	6.3%	3.5%	7.3%	6.9%
Overweight or obese (BMI 25 +)	17.2%	18.8%	18.8%	8.9%	31.9%	10.7%	18.3%	7.9%	10.2%	24.8%	14.4%	23.4%	23.3%	20.7%	7.5%	32.3%	21.2%	27.2%	23.7%
Missing	3.1%	20.4%	6.7%	11.2%	0.2%	0%	0%	4.0%	0%	0%	1.8%	2.8%	0.9%	1.3%	0%	10.5%	0.3%	0.2%	0.2%
**WHO SDS z-BMI (standard deviation score)**																			
Mean	0.131	0.312	0.032	-0.271	0.578	-0.152	0.057	-0.089	-0.120	-0.172	0.132	0.043	0.345	0.410	-1.158	0.630	0.198	0.430	0.477
Std Dev	1.294	1.229	1.524	1.130	1.263	0.977	0.879	1.014	1.061	1.699	1.054	1.496	1.072	1.072	1.617	1.025	1.013	1.256	1.077
**CDC SDS z-BMI (standard deviation score)**																			
Mean	0.051	0.226	-0.077	-0.300	0.442	-0.189	0.010	-0.003	-0.155	-0.282	0.084	-0.034	0.277	0.306	-1.263	0.503	0.130	0.329	0.380
Std Dev	1.203	1.133	1.395	1.047	1.137	0.872	0.779	0.925	0.984	1.580	0.981	1.359	0.944	0.940	1.732	0.900	0.952	1.117	0.929
**Sedentary time, total per valid day (min.day**^**−1**^**)**																			
Mean	531.8	506.3	517.9	504.2	508.7	470.2	536.8	498.1	544.1	529.3	519.6	562.7	517.0	496.3	504.5	521.2	631.6	535.8	553.5
Std Dev	88.1	78.8	98.4	76.0	93.7	100.0	95.5	79.6	80.4	83.5	76.2	89.6	81.8	73.0	78.9	63.7	69.5	82.7	78.7
**Sedentary time total during school per valid day (min.day**^**−1**^**)**																			
Mean	250.1	238.3	158.1	260.2	177.9	216.8	267.7	255.8	310.6	288.1	225.4	223.1	249.3	230.6	234.8	285.3	312.9	238.8	234.9
Std Dev	64.5	38.4	48.9	43.7	69.6	49.9	62.3	38.0	48.7	57.1	58.9	61.4	42.4	38.5	58.7	60.1	37.5	42.6	43.7
**Sedentary time total non-school per valid day (min.day**^**−1**^**)**																			
Mean	341.4	315.7	392.4	302.0	374.4	306.2	363.7	472.0	309.1	311.5	319.8	389.2	324.6	319.4	325.0	310.3	417.6	353.2	357.5
Std Dev	81.4	73.8	82.3	70.7	87.5	84.6	73.3	91.1	77.2	70.6	68.4	71.4	68.5	60.9	75.2	70.8	64.5	75.0	71.1
**MVPA, total per valid day (min.day**^**−1**^**)**																			
Mean	41.3	47.4	32.7	38.9	37.3	60.0	55.9	45.1	36.4	26.7	49.1	26.6	55.3	60.4	50.0	53.0	46.2	37.2	37.8
Std Dev	22.6	22.1	26.3	16.8	23.2	25.6	19.8	19.4	18.0	17.1	18.9	17.2	23.5	23.2	29.5	22.7	20.2	19.6	19.0
**MVPA, total during school per valid day (min.day**^**−1**^**)**																			
Mean	16.3	22.3	8.9	19.9	11.9	18.0	18.4	25.0	15.7	11.0	18.6	9.9	22.0	24.6	14.4	29.4	14.6	12.8	14.4
Std Dev	11.2	11.9	8.5	11.1	12.2	14.3	9.9	13.3	9.4	8.4	9.6	7.0	11.1	9.8	10.3	11.6	9.0	8.4	8.8
**MVPA total non-school per valid day (min.day**^**−1**^**)**																			
Mean	28.8	29.7	25.5	23.7	28.3	46.9	43.9	26.0	24.5	18.3	29.6	18.9	35.4	41.6	39.0	31.3	36.2	27.5	27.1
Std Dev	18.1	16.4	21.6	12.5	19.6	23.1	16.7	14.5	13.7	12.7	16.1	13.3	18.6	19.9	24.4	18.5	18.3	16.2	15.3
**Valid days of accelerometer wear time**																			
Mean	7.0	6.9	7.1	7.2	6.7	6.2	6.5	6.2	7.0	7.1	7.2	7.0	7.3	7.1	6.9	6.9	6.3	7.3	7.3
Std Dev	1.3	1.2	0.7	1.4	1.1	1.2	1.2	1.6	1.8	1.0	0.9	1.3	0.9	1.0	1.1	1.1	1.3	1.3	1.5
**Average valid minutes per day of accelerometer wear time**																			
Mean	807.6	786.1	843.9	797.4	806.8	794.6	804.9	774.3	776.3	817.2	804.1	820.7	809.1	808.0	829.0	795.5	860.3	813.6	799.3
Std Dev	78.3	74.1	73.4	71.2	85.1	79.9	92.9	72.2	79.0	69.3	67.0	95.0	60.4	60.1	92.2	63.0	63.3	76.6	70.8
**Average accelerometer wear time during school (minutes), total per valid day**																			
Mean	363.0	361.7	256.1	401.4	273.0	347.2	362.7	394.9	428.3	421.5	343.6	315.8	377.1	373.1	340.5	423.0	395.0	340.9	337.1
Std Dev	70.2	28.9	59.8	39.3	97.1	38.9	56.9	26.5	40.9	41.0	73.5	76.2	17.7	16.7	61.3	72.6	39.3	35.9	38.9
**Average accelerometer wear time total non-school school (minutes), total per valid day**																			
Mean	531.0	497.4	641.2	486.4	601.0	532.7	572.2	472.0	452.0	498.4	499.2	575.4	517.9	521.9	568.8	482.6	590.0	553.0	547.3
Std Dev	99.9	86.6	72.8	88.7	108.4	95.6	89.5	91.1	93.1	88.0	89.3	81.5	67.6	64.8	109.3	88.4	68.2	86.2	82.0
**ActiGraph model-filter comparable to GT3X + _LFE**																			
Comparable	92.4%	100%	100%	100%	100%	100%	100%	0%	100%	51.1%	100%	100%	100%	100%	100%	100%	100%	99.5%	78.3%

Abbreviations: *AUS* Australia, *BGD* Bangladesh, *BEL* Belgium, *BRA* Brazil, *CZE* Czech Republic, *DNK* Denmark, *HKG* Hong Kong, *IND* India, *ISR* Israel, *MYS* Malaysia, *NZL* New Zealand, *NGA* Nigeria, *PRT* Portugal, *ESP* Spain, *USA* United States of America, *IOTF* International Obesity Task Force, *BMI* Body Mass Index, *WHO* World Health Organization, *CDC* Centers for Disease Control and Prevention, *MVPA* Moderate-to-vigorous physical activity

Valid accelerometer wear time per day =  ≥ 8 h

### Socio-demographic correlates of weight status

Study site was a significant correlate of WHO BMI-SD scores and BMI categories (Table 2). For example, when compared to adolescents in Seattle (USA), adolescents in Gombe (NGA), Ghent (BEL), Olomouc (CZE), Odense (DNK), Hong Kong (CHN) and Haifa (ISR) had lower BMI-SD scores and lower odds of being overweight/obese. In contrast, adolescents from Portuguese sites and Curitiba (BRA) were more likely to be overweight/obese than adolescents from Seattle (USA). Other socio-demographic predictors were adolescent sex, educational attainment and area-level SES: Females, adolescents from a household with a college degree, and higher SES area adolescents were less likely to be overweight/obese.

**Table 2:** Associations of socio-demographic variables, study site, and neighbourhood characteristics with WHO BMI-SD and BMI categories (IOTF)

**Socio-demographic variable, study site & neighbourhood characteristics**	**WHO BMI-SD**		**BMI categories (IOTF)** **(thin/normal vs. overweight/obese)**	
	***b* (95% CIs)**	***p*-value**	**OR (95% CIs)**	***p*-value**
Sex (ref: male)				
Female	-0.072 (-0.144, 0.0003)	.051	0.747 (0.644, 0.867)	< .001
Age, years	-0.014 ( -0.038, 0.011)	.278	1.015 (0.964, 1.069)	.561
City (ref: Seattle)				
Baltimore, USA	-0.036 (-0.203, 0.131)	.673	1.234 (0.894, 1.702)	.201
Gombe, NGA	-1.640 (-1.840, -1.438)	< .001	0.242 (0.140, 0.417)	< .001
Ghent, BEL	-0.691 (-0.897, -0.485)	< .001	0.430 (0.255, 0.726)	.002
Valencia, ESP	-0.253 (-0.440, -0.065)	.008	0.804 (0.549, 1.177)	.261
Porto region, PRT	0.098 (-0.150, 0.346)	.440	1.633 (1.036, 2.572)	.035
Olomouc, CZE	-0.598 (-0.947, -0.248)	< .001	0.403 (0.163, 0.997)	.049
Hradec Králové, CZE	-0.373 (-0.749, 0.003)	.052	0.723 (0.319, 1.638)	.437
Odense, DNK	-0.563 (-0.819, -0.306)	< .001	0.327 (0.162, 0.657)	.002
Curitiba, BRA	0.090 (-0.082, 0.262)	.304	1.426 (1.029, 1.977)	.033
Kuala Lumpur, MYS	-0.423 (-0.626, -0.221)	< .001	0.943 (0.603, 1.475)	.798
Melbourne, AUS	-0.175 (-0.358, 0.007)	.059	0.972 (0.670, 1.410)	.881
Auckland, NZL	-0.120 (-0.301, 0.061)	.195	0.980 (0.686, 1.401)	.913
Wellington, NZL	-0.075 (-0.305, 0.155)	.522	0.783 (0.493, 1.244)	.300
Hong Kong, CHN	-0.588 (-0.754, -0.422)	< .001	0.355 (0.240, 0.526)	< .001
Dhaka, BGD	-0.465 (-0.766, -0.163)	.003	0.773 (0.399, 1.496)	.444
Chennai, IND	-0.658 (-0.841, -0.474)	< .001	1.020 (0.713, 1.457)	.915
Haifa, ISR	-0.317 (-0.525, -0.108)	.003	0.559 (0.352, 0.887)	.014
Education (ref: < college)				
College or higher	-0.028 (-0.108, 0.051)	.485	0.826 (0.702, 0.970)	.020
Walkability (ref: low)				
High	-0.049 (-0.122, 0.025)	.193	0.948 (0.812, 1.105)	.492
SES (ref: low)				
High	-0.105 (-0.179 -0.031)	.005	0.846 (0.722, 0.990)	.037

Abbreviations: *AUS* Australia, *BGD* Bangladesh, *BEL* Belgium, *BRA* Brazil, *CZE* Czech Republic, *DNK* Denmark, *CHN* China, *IND* India, *ISR* Israel, *MYS* Malaysia, *NZL* New Zealand, *NGA* Nigeria, *PRT* Portugal, *ESP* Spain, *USA* United States of America, *SES* area-level socio-economic status, *IOTF* International Obesity Task Force, *BMI* Body Mass Index, *WHO* World Health Organization, *OR* Odds ratio, *CI* Confidence interval. Estimates from generalized additive mixed models with random intercepts at the administrative-unit and school levels (pooled estimates from 10 imputed datasets)

### Main associations of accelerometer-based MVPA and ST with BMI and weight status (study aim 1)

Total MVPA and ST were both negatively related to adolescent BMI-SD scores and the likelihood of being overweight/obese (Table 3). For every 10 min/day increase in total MVPA, BMI-SD decreased by 0.04 SD units. Similarly, for every 10 min/day increase in total ST, BMI-SD decreased by 0.01 SD units. When examining the context-specific effects of MVPA and ST (Model 2 in Table 3), ST during school periods and MVPA during non-school periods were more strongly negatively related to BMI-SD scores and the odds of being overweight/obese than their counterpart measures.

**Table 3:** Associations of time spent in MVPA and ST with WHO BMI-SD and BMI categories (IOTF): main effect models

**MVPA / ST variables**	**WHO BMI-SD**		**BMI categories (IOTF) (thin/normal vs. overweight/obese)**	
	***b* (95% CIs)**	***p*-value**	**OR (95% CIs)**	***p*-value**
*Model 1: total MVPA/ST*				
Total MVPA (min/day)	-0.004 (-0.006, -0.002)	< .001	0.989 (0.985, 0.994)	< .001
Total ST (min/day)	-0.001 (-0.002 -0.0006)	< .001	0.997 (0.995, 0.998)	< .001
*Model 2: School and non-school MVPA/ST*				
School MVPA (min/day)	-0.004 (-0.009, 0.001)	.120	0.990 (0.980, 1.000)	.062
School ST (min/day)	-0.002 (-.003, -0.0003)	.016	0.996 (0.993, 0.999)	.004
Non-School MVPA (min/day)	-0.003 (-0.006, -0.001)	.017	0.991 (0.985, 0.997)	.003
Non-School ST (min/day)	-0.0008 (-.0021, 0.0003)	.131	0.999 (0.996, 1.000)	.093

Abbreviations: *MVPA* Moderate-to-vigorous physical activity, *ST* Sedentary time, *IOTF* International Obesity Task Force, *BMI* Body Mass Index, *WHO* World Health Organization, *OR* Odds ratio, *CI* Confidence interval. Estimates from generalized additive mixed models with random intercepts at the administrative-unit and school levels (pooled estimates from 10 imputed datasets). Models were adjusted for adolescent sex, age, city, area-level walkability and SES, valid days of accelerometer wear, average wear time per day and accelerometer comparability

### Moderating effects of study site, sex (study aim 2) and accelerometer comparability (sensitivity analyses)

Some associations of MVPA and ST measures with adolescents’ BMI outcomes were moderated by study site or accelerometer comparability, while adolescent sex was not a significant moderator (all *p* values > 0.450) (Table 4). Site moderated the association of total MVPA with BMI-SD score, with adolescents in Baltimore (USA), Seattle (USA), Ghent (BEL), Gombe (NGA), Chennai (IND), Wellington (NZL), Curitiba (BRA), Melbourne (AUS), and Olomouc (CZE) tending to show a negative association; adolescents in Kuala Lumpur (MYS) and Valencia (ESP) tending to show a positive association; and those in other study sites close to null association. Study site also moderated the relation of MVPA during non-school periods to BMI-SD scores. The pattern was similar to that of total MVPA with BMI-SD scores.

**Table 4:** Associations of MVPA and ST with WHO BMI-SD: full models including moderating effects

Model 1: Total MVPA/ST			Model 2: School and non-school MVPA/ST		
**Regression term**	***b* (95% CIs)**	***p*-value**	**Regression term**	***b* (95% CIs)**	***p*-value**
			MVPA during school (main effect)	-0.004 (-0.008, 0.001)	.154
			ST during school (main effect)	-0.002 (-0.003, -0.0004)	.010
*City-specific effects of total MVPA*			*City-specific effects of non-school MVPA*		
Seattle, USA	-0.006 (-0.012, 0.001)	.091	Seattle, USA	-0.006 (-0.014, 0.002)	.139
Baltimore, USA	-0.009 (-0.015, -0.003)	.003	Baltimore, USA	-0.010 (-0.017, -0.002)	.009
Gombe, NGA	-0.015 (-0.021, -0.010)	< .001	Gombe, NGA	-0.017 (-0.024, -0.010)	< .001
Ghent, BEL	-0.006 (-0.017, 0.004)	.242	Ghent, BEL	-0.0002, (-0.014, 0.014)	.975
Valencia, ESP	0.004 (-0.002, 0.011)	.170	Valencia, ESP	0.006 (-0.001, 0.013)	.105
Porto region, PRT	0.002 (-0.008, 0.012)	.704	Porto region, PRT	0.003 (-0.009, 0.016)	.605
Olomouc, CZE	-0.008 (-0.021, 0.004)	.199	Olomouc, CZE	-0.009 (-0.023, 0.005)	.219
Hradec Králové, CZE	0.002 (-0.015, 0.020)	.786	Hradec Králové, CZE	0.004 (-0.016, 0.025)	.681
Odense, DNK	-0.003 (-0.015, 0.009)	.621	Odense, DNK	-0.002 (-0.018, 0.014	.804
Curitiba, BRA	-0.004 (-0.010, 0.001)	.106	Curitiba, BRA	-0.003 (-0.009, 0.004)	.430
Kuala Lumpur, MYS	0.010 (0.002, 0.019)	.016	Kuala Lumpur, MYS	0.015 (0.003, 0.026)	.014
Melbourne, AUS	-0.004 (-0.010, 0.003)	.247	Melbourne, AUS	-0.004 (-0.013, 0.005)	.394
Auckland, NZL	-0.001 (-0.007, 0.005)	.701	Auckland, NZL	-0.001 (-0.008, 0.006)	.815
Wellington, NZL	-0.006 (-0.014, 0.002)	.158	Wellington, NZL	-0.008 (-0.018, 0.002)	.100
Hong Kong, CHN	0.002 (-0.004, 0.008)	.532	Hong Kong, CHN	0.003 (-0.004, 0.011)	.400
Dhaka, BGD	-0.001 (-0.011, 0.010)	.886	Dhaka, BGD	-0.001 (-0.014, 0.012)	.879
Chennai, IND	-0.012 (-0.020, -0.003)	.007	Chennai, IND	-0.015 (-0.026, -0.004)	.009
Haifa, ISR	-0.001 (-0.010, 0.008)	.806	Haifa, ISR	0.001 (-0.010, 0.011)	.860
*Accelerometer-comparability-specific effects of total ST*			*Accelerometer-comparability-specific effects of non-school ST*		
Not comparable accelerometers	0.001 (-0.001, 0.003)	.208	Not comparable accelerometers	0.002 (-0.0002, 0.004)	.087
Comparable accelerometers	-0.001 (-0.002, -0.001)	< .001	Comparable accelerometers	-0.001 (-0.002, 0.0003)	.140

Abbreviations: *AUS* Australia, *BGD* Bangladesh, *BEL* Belgium, *BRA* Brazil, *CZE* Czechia, *DNK* Denmark, *CHN* China, *IND* India, *ISR* Israel, *MYS* Malaysia, *NZL* New Zealand, *NGA* Nigeria, *PRT* Portugal, *ESP* Spain, *USA* United States of America, *MVPA* Moderate-to-vigorous physical activity, *ST* Sedentary time, *BMI* Body Mass Index, *WHO* World Health Organization, *OR* Odds ratio, *CI* Confidence interval. Estimates from generalized additive mixed models with random intercepts at the administrative-unit and school levels (pooled estimates from 10 imputed datasets). Models were adjusted for adolescent sex, age, city, area-level walkability and SES, valid days of accelerometer wear, average wear time per day and accelerometer comparability

Associations between total ST and BMI-SD score were moderated by accelerometer comparability, with negative associations observed only in adolescents wearing comparable accelerometers. The full model of context-specific MVPA and ST including significant moderating effects indicated that ST during school periods was similarly negatively related to BMI-SD score across sexes, study sites and accelerometer models. In contrast, the associations of ST during non-school periods with BMI-SD scores were moderated by accelerometer comparability, whereby non-comparable accelerometers tended to yield positive, and comparable accelerometers negative, associations (Table 4).

Accelerometer comparability also moderated the associations of total ST and ST during non-school periods with the likelihood of being overweight/obese, whereby only comparable accelerometers tended to show a negative association (Table 5). The remaining MVPA and ST measures were negatively related with the odds of being overweight/obese, although the association with MVPA during school periods was weak. These effects were not moderated by study site, sex or accelerometer comparability.

**Table 5:** Associations of MVPA and ST with BMI categories (IOTF) (thin/normal vs. overweight/obese): full models including moderating effects

Model 1: Total MVPA/ST			Model 2: School and non-school MVPA/ST		
**Regression term**	**OR (95% CIs)**	***p*-value**	**Regression term**	**OR (95% CIs)**	***p*-value**
Total MVPA (min/day; main effect)	0.990 (0.985, 0.994)	< .001	MVPA during school (min/day; main effect)	0.990 (0.980, 1.001)	.066
			Non-school MVPA (min/day; main effect)	0.996 (0.993, 0.999)	.004
			ST during school (min/day; main effect)	0.991 (0.985, 0.997)	.003
*Accelerometer-comparability-specific effects of total ST (min/day)*			*Accelerometer-comparability-specific effects of non-school ST (min/day)*		
Not comparable accelerometers	1.001 (0.997, 1.005)	.696	Not comparable accelerometers	1.002 (0.998, 1.006)	.330
Comparable accelerometers	0.997 (0.995, 0.998)	< .001	Comparable accelerometers	0.998 (0.996, 1.000)	.055

Abbreviations: *ITOF* International Obesity Task Force, *MVPA* Moderate-to-vigorous physical activity, *ST* Sedentary time, *OR* Odds ratio, *CI* Confidence interval. Estimates from generalized additive mixed models with random intercepts at the administrative-unit and school levels (pooled estimates from 10 imputed datasets). Models were adjusted for adolescent sex, age, city, area-level walkability and SES, valid days of accelerometer wear, average wear time per day and accelerometer comparability

Similar findings were observed when analyses were conducted on cases with complete data and when using CDC BMI-SD scores as outcome variables (sensitivity analyses; Supplementary material: Appendices 1,2,3,4).

## Discussion

The most notable finding was that associations between MVPA and sex- and age-adjusted weight status outcomes were stronger and more robust than associations found for ST. This is in line with previous research in adolescents [7, 8, 32, 33]. Remarkably and unexpectedly, most associations between ST variables and weight outcomes were negative, implying that more ST was associated with lower BMI-SD scores and lower odds of being overweight/obese. To our knowledge, almost no previous studies reported such negative associations. A scientific statement of the American Heart Association concluded that, based on cross-sectional and longitudinal data, little to no association between objectively-measured sedentary time and markers of adiposity (e.g., BMI) in adolescents is present [34]. A recently published scoping review focusing exclusively on ActiGraph studies that examined associations of PA and ST with body composition in children and adolescents concluded that ST was positively associated with BMI and percentage fat, and negatively with lean mass [33]. However, in that scoping review, data from children and adolescents were combined, and all included studies were conducted in Western Europe, North America, Brazil or Africa. No data were included of Asian adolescents who usually have a lower muscle mass than their Caucasian counterparts [35]. None of these studies, including ours, examined sleep time, a known modulator of obesity [36].

Our findings indicated the negative associations of overall and non-school-based ST with adjusted weight outcomes were present in adolescents wearing comparable accelerometers (92.4% of the sample). These negative associations might be partially explained by a compensation mechanism. Adolescents engaging in vigorous PA (and thus having a lower BMI) might be more sedentary during the rest of the day. Although our analyses mutually adjusted for MVPA and ST there might have been residual confounding of vigorous PA. The correlations between ST and vigorous PA support this hypothesis. In several study sites using comparable accelerometers, there were positive associations between ST and vigorous PA, mainly in male participants (e.g., *r* = 0.30 in India, *r* = 0.35 in Bangladesh, *r* = 0.57 in Olomouc). Positive associations between non-school-based ST and BMI-SD score were found in adolescents wearing non-comparable accelerometers, but this subsample only consisted of 370 adolescents from Denmark, India and USA.

Accelerometer-based methodological issues clearly play an important role in data analyses and results interpretation. While our study took extra steps to harmonize study procedures and measures across countries (e.g., scoring accelerometer data at one coordinating centre; fewer than 8% of participants wore non-comparable accelerometers), the importance of procedural uniformity should be underscored for future studies. Multi-country studies involving accelerometer data pooling should emphasize the importance of using identical data collection procedures (e.g. use of LFE and comparable ActiGraph models), using uniform scoring procedures, and being transparent in reporting data collection and processing procedures in manuscripts. These practices should enhance the possibility of comparing findings across sites and avoiding methodological biases.

A second key finding was the country-specificity of associations between MVPA and adjusted weight outcomes. In most sites, negative associations were found, but in some (Porto region (PRT), Dhaka (BGD), Haifa (ISR)) no associations or even a tendency towards positive associations (Valencia (ESP), Kuala Lumpur (MYS)) were identified. These cross-site differences support the importance of conducting multi-country studies. Differences in associations between MVPA and weight outcomes between countries could depend on interactions between cultural and biological factors, like differences in body composition across ethnic groups, differences in types of activities adolescents engage in across countries (e.g. cycling or water-based activities that are not captured by accelerometers), or differences in diet.

A novel aspect was the examination of school-based and non-school-based MVPA and ST separately as correlates of weight status outcomes. Non-school-based MVPA and school-based ST were more strongly negatively related to BMI-SD scores and BMI categories than school-based MVPA and non-school-based ST. Almost no previous studies examined these context-specific associations. One study using a compositional isotemporal substitution model found that replacing 30 min of out-of-school ST with 30 min out-of-school light-intensity PA was associated with a decrease in adiposity, while no such associations were found for school-based ST [37]. A systematic review concluded there was insufficient evidence to suggest health benefits may differ depending on PA context (e.g. recreation versus active transportation versus school-based) [38]. They emphasized the need to address this knowledge gap in future research, to be able to provide more specificity in PA guidelines. A potential explanation for our findings might be the higher variability in the amount of non-school-based PA compared with school-based PA, which is also reflected in the descriptive statistics.

None of the detected associations of MVPA and ST with weight outcomes were curvilinear. This is not in line with findings of the IPEN Adult study, a multi-country study with similar methods and analyses [11]. In adults from 10 countries, curvilinear associations between MVPA and weight outcomes were found, with an attenuation of the beneficial effect of MVPA on BMI at higher levels of MVPA [11]. However, in adolescents, it seems like higher levels of MVPA are more beneficial for weight outcomes, without evidence of a ‘levelling-off’ effect. This finding supports the recently updated WHO guidelines on PA for children and adolescents, stating that children and adolescents should do *at least* an average of 60 min per day of MVPA across the week [39]. In a review to inform the updated WHO guidelines, it was reported that more PA beyond a daily 60 min of MVPA appears to be better for several health outcomes [38].

Overall, this paper adds novelty to the current evidence base by examining potential curvilinearity in the associations, including data from Asian and African countries and focusing on school- and non-school-based PA and ST, in addition to total volumes of PA and ST. Our results indicate that behavior-specific and site-specific recommendations and interventions may be more promising than broad recommendations to reduce ST and increase MVPA. Furthermore, as no moderating effects of adolescent sex were found, interventions could be expected to be similarly effective for girls and boys.

The primary study strength is the comparable data collection protocols in 15 diverse countries across six continents, including regions that are currently underrepresented in PA research (e.g., Malaysia, Bangladesh, India, Nigeria). Other strengths are the large overall sample size, application of GAMMs that allowed modelling of curvilinear associations, and use of accelerometers. Several limitations need acknowledgement. First, the cross-sectional design precluded inferences about causality. Second, estimates of MVPA and ST are probably not representative of the total adolescent population in participating countries, as participants were purposively recruited from specific neighbourhoods. Third, ActiGraph models used varied, as did response rates across sites, implying sampling biases or other methodological biases. Fourth, waist-worn accelerometers do not specifically measure sedentary time or changes in posture; they measure lack of movement. Consequently, time spent standing can be incorrectly classified as sedentary time when using waist-worn accelerometers. Fifth, a combination of self-report and objective measures of weight and height was used across countries. Objectively-assessed BMI has superior accuracy to classify adolescent participants as overweight or obese [40]. Sixth, ideally, diet-related measures and information on sleep duration/quality would have been included as well. Furthermore, stage of maturity plays an important role in the development of BMI, so it would have been useful if we could have controlled for this in the analyses. However, no such information was collected. Finally, there were large differences in sample sizes across countries, limiting the power in country-specific analyses.

## Conclusions

In conclusion, this international study confirmed prior research that MVPA may be a stronger correlate of weight status than ST. More specifically, non-school-based MVPA was more strongly related to weight status than school-based MVPA, but this should be examined further in future studies. Surprisingly, more ST was related to lower BMI and lower odds of being overweight/obese, although this result was not consistent across study sites. This finding should be further explored in other methodologically sound international studies to determine if present results can be replicated. Finally, we encourage the use of inclinometers or pressure sensors (e.g. in socks or shoes) [41] to provide more precise measures of ST.

## Supplementary Information

**Additional file 1.**

**Additional file 2.** 

**Additional file 3.**

**Additional file 4.**

**Additional file 5.**

## Data Availability

The dataset supporting the conclusions of this article is available upon request, by emailing to the corresponding author. Because of the international nature of the study, and different data sharing rules in the different institutions, the data cannot be made readily available.

## Abbreviations

BMI: Body mass index
PA: Physical activity
ST: Sedentary time
MVPA: Moderate-to-vigorous physical activity
GAMMS: Generalized additive multilevel mixed models
SD: Standard deviation

## References

[1] NCD Risk Factor Collaboration (NCD-RisC) (2017). Worldwide trends in body-mass index, underweight, overweight, and obesity from 1975 to 2016: a pooled analysis of 2416 population-based measurement studies in 128.9 million children, adolescents, and adults. Lancet.

[2] WHO (2016). Consideration of the evidence on childhood obesity for the Commission on Ending Childhood Obesity: report of the Ad hoc Working Group on Science and Evidence for Ending Childhood Obesity.

[3] Park M, Falconer C, Viner R, Kinra S (2012). The impact of childhood obesity on morbidity and mortality in adulthood: a systematic review. Obes Rev.

[4] Liang J, Matheson BE, Kaye WH, Boutelle KN (2014). Neurocognitive correlates of obesity and obesity-related behaviors in children and adolescents. Int J Obes (Lond).

[5] Quek YH, Tam WWS, Zhang MWB, Ho RCM (2017). Exploring the association between childhood and adolescent obesity and depression: a meta-analysis. Obes Rev.

[6] Ross SE, Flynn JI, Pate RR (2016). What is really causing the obesity epidemic? A review of reviews in children and adults. J Sports Sci.

[7] Tarp J, Child A, White T (2018). Physical activity intensity, bout-duration, and cardiometabolic risk markers in children and adolescents. Int J Obes.

[8] Crowe M, Sampasa-Kanyinga H, Saunders TV, Hamilton HA, Benchimol EI, Chaput JP (2020). Combinations of physical activity and screen time recommendations and their association with overweight/obesity in adolescents. Can J Public Health.

[9] Kwon S, Ekelund U, Kandula N, Janz KF (2022). Joint associations of physical activity and sedentary time with adiposity during adolescence: ALSPAC. Eur J Public Health..

[10] Skrede T, Steene-Johannessen J, Anderssen SA, Resaland GK, Ekelund U (2019). The prospective association between objectively measured sedentary time, moderate-to-vigorous physical activity and cardiometabolic risk factors in youth: a systematic review and meta-analysis. Obes Rev.

[11] Van Dyck D, Cerin E, De Bourdeaudhuij I (2015). International study of objectively measured physical activity and sedentary time with body mass index and obesity: IPEN adult study. Int J Obes.

[12] Migueles JH, Aadland E, Andersen LB, et al. GRANADA consensus on analytical approaches to assess associations with accelerometer-determined physical behaviours (physical activity, sedentary behaviour and sleep) in epidemiological studies. Br J Sports Med 2021; e-pub ahead of print 12 April 2021; doi: 10.1136/bjsports-2020-103604. 10.1136/bjsports-2020-103604 PMC8938657 33846158

[13] Cain K, Salmon J, Conway TL (2021). International Physical Activity and Built Environment Study of Adolescents: IPEN Adolescent design, protocol and measures. BMJ Open.

[14] Frank LD, Sallis JF, Saelens BE, Leary L, Cain K, Conway TL, Hess PM (2010). The development of a walkability index: application to the neighbourhood quality of life study. Br J Sports Med.

[15] Adams MA, Frank LD, Schipperijn J (2014). International variation in neighborhood walkability, transit, and recreation environments using geographic information systems: the IPEN adult study. Int J Health Geogr.

[16] Pan H, Cole TJ. LMSgrowth, a Microsoft Excel add-in to access growth references based on the LMS method. 2012. Available on: https://www.healthforallchildren.com/lmsgrowth-download/ (access verified on September 22, 2021).

[17] de Onis M, Onyango AW, Borghi E, Siyam A, Nishida C, Siekmann J (2007). Development of a WHO growth reference for school-aged children and adolescents. Bull World Health Organ.

[18] Cole TJ, Bellizzi MC, Flegal KM, Dietz WH (2000). Establishing a standard definition for child overweight and obesity worldwide: international survey. BMJ.

[19] Cole TJ, Flegal KM, Nicholls D, Jackson AA (2007). Body mass index cut offs to define thinness in children and adolescents: international survey. BMJ.

[20] Kuczmarski RJ, Ogden CL, Guo SS (2002). 2000 CDC Growth Charts for the United States: methods and development. Vital Health Stat.

[21] Cain KL, Sallis JF, Conway TL (2013). Using accelerometers in youth physical activity studies: a review of methods. J Phys Act Health.

[22] Cain KL, Bonilla E, Conway TL (2018). Defining accelerometer nonwear time to maximize detection of sedentary time in youth. Pediatr Exerc Sci.

[23] Evenson KR, Catellier DJ, Gill K, Ondrak KS, McMurray RG (2008). Calibration of two objective measures of physical activity for children. J Sports Sci.

[24] Cain KL, Conway TL, Adams MA, Husak LE, Sallis JF (2013). Comparison of older and newer generations of ActiGraph accelerometers with the normal filter and the low frequency extension. Int J Behav Nutr Phys Act.

[25] Rubin DB (1987). Multiple Imputation for Nonresponse in surveys.

[26] Van Buuren S (2012). Flexible Imputation of Missing Data.

[27] Wood SN (2006). Generalized Additive Models: an Introduction with R.

[28] Burnham KP, Anderson DR (2002). Model selection and multimodel inference: A practical information-theoretic approach.

[29] Fox J, Weisberg S (2011). An R companion to applied regression.

[30] Warnes GR. Gmodels. Various R programming tools for model fitting. R Package version 2.15.3. 2012. http://CRAN.R-project.org/package=gmodels

[31] Van Buuren S, Groothuis-Oudshoorn K (2011). mice: Multivariate imputation by chained equations in R. J Stat Softw.

[32] Ekelund U, Luan J, Sherar L, Esliger DW, Griew P, Cooper A, or the International Children’s Accelerometry Database (ICAD) Collaborators (2012). Moderate to vigorous physical activity and sedentary time and cardiometabolic risk factors in children and adolescents. JAMA..

[33] Gualdi-Russo E, Rinaldo N, Toselli S, Zaccagni L (2021). Associations of physical activity and sedentary behaviour assessed by accelerometer with body composition among children and adolescents: a scoping review. Sustainability.

[34] Barnett TA, Kelly AS, Rohm Young D, Perry CK, Pratt CA, Edwards NM (2018). Sedentary behaviors in today’s youth: approaches to the prevention and management of childhood obesity – A scientific statement from the American Heart Association. Circulation.

[35] Wulan SN, Westerterp KR, Plaqui G (2010). Ethnic differences in body composition and the associated metabolic profile: a comparative study between Asians and Caucasians. Maturitas.

[36] Gohil A, Hannon TS (2018). Poor sleep and obesity: concurrent epidemics in adolescent youth. Front Endocrinol (Lausanne).

[37] Gaba A, Dygryn J, Stefelova N, Rubin L, Hron K, Jakubec L (2021). Replacing school and out-of-school sedentary behaviors with physical activity and its associations with adiposity in children and adolescents: a compositional isotemporal substitution analysis. Environ Health Prev Med.

[38] Chaput JP, Willumsen J, Bull F (2020). 2020 WHO guidelines on physical activity and sedentary behaviour for children and adolescents aged 5–17 years: summary of the evidence. Int J Behav Nutr Phys Act.

[39] World Health Organization. Fact sheet on physical activity, 26 November 2020. https://www.who.int/news-room/fact-sheets/detail/physical-activity, accessed on October 7, 2021.

[40] Karchynskaya V, Kopcakova J, Klein D (2020). Is BMI a valid indicator of overweight and obesity for adolescents?. Int J Environ Res Public Health.

[41] Sanders JP, Loveday A, Pearson N (2016). Devices for self-monitoring sedentary time for physical activity: a scoping review. J Med Internet Res.

