# Supplementary material for: Associations of accelerometer measured school- and non-school based physical activity and sedentary time with body mass index: IPEN Adolescent study
Source: Int J Behav Nutr Phys Act. 2022 Jul 14;19:85. doi: 10.1186/s12966-022-01324-x (PMC9284738; doi:10.1186/s12966-022-01324-x)
Supplement: Supplementary file 1 — Additional file 1. [file 12966_2022_1324_MOESM1_ESM.docx]

**Appendix 1: Associations between adolescent WHO BMI z-score and total MVPA & ST (complete cases analyses)**

**1a. Associations of socio-demographic characteristics with adolescent WHO BMI z-score**

|  | **b** | **95%CIs** | **p** |
| --- | --- | --- | --- |
| Sex (ref: male) |  |  |  |
| Female | -0.077 | (-0.151, -0.002) | 0.043* |
| Age (range 11-19 years) | -0.014 | (-0.392, 0.012) | 0.301 |
| City (ref: Seattle, USA) |  |  |  |
| Baltimore, USA | -0.035 | (-0.203, 0.133) | 0.683 |
| Gombe, NGA | -1.685 | (-1.889, -1.480) | <0.001*** |
| Ghent, BEL | -0.746 | (-0.959, -0.533) | <0.001*** |
| Valencia, ESP | -0.258 | (-0.447, -0.069) | 0.008** |
| Porto region, PRT | 0.167 | (-0.101, 0.453) | 0.222 |
| Olomouc, CZE | -0.653 | (-1.095, -0.210) | 0.004** |
| Hradec Králové, CZE | -0.469 | (-1.011, 0.073) | 0.090 |
| Odense, DNK | -0.553 | (-0.811, -0.294) | <0.001*** |
| Curitiba, BRA | 0.090 | (-0.082, 0.263) | 0.303 |
| Kuala Lumpur, MYS | -0.434 | (-0.658, -0.211) | <0.001*** |
| Melbourne, AUS | -0.157 | (-0.383, 0.069) | 0.173 |
| Auckland, NZL | -0.117 | (-0.304, 0.070) | 0.218 |
| Wellington, NZL | -0.089 | (-0.323, 0.145) | 0.457 |
| Hong Kong, CHN | -0.586 | (-0.755, -0.147) | <0.001*** |
| Dhaka, BGD | -0.472 | (-0.786, -0.159) | 0.003** |
| Chennai, IND | -0.664 | (-0.847, -0.480) | <0.001 |
| Haifa, ISR | -0.303 | (-0.514, -0.093) | 0.005** |
| Education (ref: <college) |  |  |  |
| ≥college | -0.033 | (-0.113, 0.046) | 0.414 |
| Walkability (ref: low) |  |  |  |
| High | -0.053 | (-0.130, 0.023) | 0.173 |
| SES (ref: low) |  |  |  |
| High | -0.105 | (-0.183, -0.027) | 0.008** |

**Notes:** Abbreviations: AUS, Australia; BGD, Bangladesh; BEL, Belgium; BRA, Brazil; CZE, Czechia; DNK, Denmark; CHN, China; IND, India; ISR, Israel; MYS, Malaysia; NZL, New Zealand; NGA, Nigeria; PRT, Portugal; ESP, Spain; USA, United States of America; MVPA, moderate-to-vigorous physical activity time; ST, sedentary time; SES, area-level socio-economic status; * = p<0.05; ** = p<0.01; *** = p<0.001

**Conclusion:** Female adolescents were likely to have lower WHO BMI z-score values. There was no significant association of WHO BMI z-score values with adolescent age. No cities had significantly higher average WHO BMI z-score values than the reference city (Seattle USA), while average WHO BMI z-score values in seven cities were not significantly different from Seattle and in eleven cities were significantly lower than in Seattle. On average, adolescents living in high walkability and/or high SES areas had lower WHO BMI z-score values than their counterparts.

**1b. Associations of socio-demographic characteristics with adolescent CDC BMI Z-score (sensitivity analysis)**

|  | **b** | **95%CIs** | **P** |
| --- | --- | --- | --- |
| Sex (ref: male) |  |  |  |
| Female | 0.022 | (-0.046, 0.0908) | 0.526 |
| Age (range 11-19 years) | -0.012 | (-0.036, 0.011) | 0.301 |
| City (ref: Seattle, USA) |  |  |  |
| Baltimore, USA | -0.047 | (-0.201, 0.108) | 0.556 |
| Gombe, NGA | -1.682 | (-1.870, -1.494) | <0.001 *** |
| Ghent, BEL | -0.689 | (-0.885, -0.492) | <0.001 *** |
| Valencia, ESP | -0.236 | (-0.410, -0.063) | 0.008** |
| Porto region, PRT | 0.062 | (-0.086, 0.409) | 0.201 |
| Olomouc, CZE | -0.604 | (-1.012, -0.196) | 0.004 ** |
| Hradec Králové, CZE | -0.434 | (-0.932, 0.065) | 0.088 |
| Odense, DNK | -0.495 | (-0.732, -0.258) | <0.001 *** |
| Curitiba, BRA | 0.054 | (-0.104, 0.213) | 0.503 |
| Kuala Lumpar & others, MYS | -0.423 | (-0.624, -0.223) | <0.001 *** |
| Melbourne, AUS | -0.156 | (-0.364, 0.053) | 0.143 |
| Auckland, NZL | -0.100 | (-0.272, 0.073) | 0.257 |
| Wellington, NZL | -0.063 | (-0.279, 0.154) | 0.570 |
| Hong Kong, CHN | -0.533 | (-0.687, -0.378) | <0.001 *** |
| Dhaka, BGD | -0.480 | (-0.765, -0.195) | <0.001 *** |
| Chennai, IND | -0.672 | (-0.842, -0.503) | <0.001 *** |
| Haifa, ISR | -0.268 | (-0.461, -0.075) | 0.007 ** |
| Education (ref: <college) |  |  |  |
| ≥college | -0.017 | (-0.090, 0.057) | 0.655 |
| Walkability (ref: low) |  |  |  |
| High | -0.053 | (-0.123, 0.017) | 0.140 |
| SES (ref: low) |  |  |  |
| High | -0.090 | (-0.161, -0.019) | 0.013 * |

**Notes:** Abbreviations: AUS, Australia; BGD, Bangladesh; BEL, Belgium; BRA, Brazil; CZE, Czechia; DNK, Denmark; CHN, China; IND, India; ISR, Israel; MYS, Malaysia; NZL, New Zealand; NGA, Nigeria; PRT, Portugal; ESP, Spain; USA, United States of America; MVPA, moderate to vigorous physical activity time, ST, sedentary time, area-level socio-economic status and SES; * = p<0.05; ** = p<0.01; *** = p<0.001

**2a. Linear main effects of total MVPA and ST on adolescent WHO BMI z-score (the best main effects model)**

|  | **b** | **95%CIs** | **p** |
| --- | --- | --- | --- |
| MVPA (min/day) | -0.004 | (-0.006, -0.002) | <0.001*** |
| ST (min/day) | -0.001 | (-0.002, -0.0004) | <0.001*** |

**Notes:** Model adjusted for adolescent sex, age, city, area-level walkability and SES, valid days of accelerometer wear, average wear time per day and accelerometer comparability; MVPA, moderate-to-vigorous physical activity time; ST, sedentary time; *** = p<0.001

**Conclusion:** WHO BMI z-score was lower in adolescents with higher levels of MVPA and/or ST during valid accelerometer wear days. No evidence of curvilinear effects was found.

**2b. Linear main effects of total MVPA and ST on adolescent CDC BMI z-score (sensitivity analysis)**

|  | **b** | **95%CIs** | **p** |
| --- | --- | --- | --- |
| MVPA (min/day) | -0.003 | (-0.005, -0.001) | <0.001*** |
| ST (min/day) | -0.001 | (-0.002, -0.0003) | <0.001*** |

**Notes:** Model adjusted for adolescent sex, age, city, area-level walkability and SES, valid days of accelerometer wear, average wear time per day and accelerometer comparability; MVPA, moderate-to-vigorous physical activity time; ST, sedentary time; *** = p<0.001

**Conclusion:** CDC BMI z-score was lower in adolescents with higher levels of MVPA and/or ST during valid accelerometer wear days. Thus, the findings for WHO BMI and CDC BMI z-scores did not differ.

**3a. Accelerometer comparability as a moderator of the effects of total MVPA and ST on adolescent WHO BMI z-score (i.e., do the main effects depend on the accelerometers used?)**

| **Regression terms** | **Estimate** | **95% CI** | **p** |
| --- | --- | --- | --- |
| **Model of moderating effects of accelerometer comparability with total MVPA and ST** |  |  |  |
| MVPA:Accelerometer comparability (interaction term) | 0.006 | (-0.002, 0.014) | 0.148 |
| ST:Accelerometer comparability (interaction term) | -0.002 | (-0.004, -0.0004) | 0.020* |
| **Model of moderating effects of accelerometer comparability with total ST and main effect of total MVPA** |  |  |  |
| MVPA (main effect) | -0.004 | (-0.006, -0.001) | <0.001*** |
| ST:Accelerometer comparability (interaction term) | -0.003 | (-0.005, -0.001) | <0.001*** |
| ***Accelerometer-comparability-specific effects of total ST*** |  |  |  |
| ST in those with non-comparable accelerometer | 0.0015 | (-0.0002, 0.003) | 0.088. |
| ST in those with comparable accelerometer | -0.0015 | (-0.002, -0.001) | <0.001*** |

**Notes:** Model adjusted for adolescent sex, age, city, area-level walkability and SES, valid days of accelerometer wear, average wear time per day and accelerometer comparability; ST, sedentary time; * = p<0.05; ** = p<0.01; *** = p<0.001

**Conclusion:** Accelerometry comparability did not determine the effect of MVPA on WHO BMI z-score, however, it moderated the effect of ST on WHO BMI z-score. For participants who did not have comparable accelerometers, the association between ST & WHO BMI z-score was not significant but it tended to be positive. In contrast for participants who wore comparable accelerometers (i.e., comparable to GT3X+_LFE), the association between ST & WHO BMI z-score was negative.

**3b. Accelerometer comparability as a moderator of the effects of total MVPA and ST on adolescent CDC BMI z-score (i.e., do the main effects depend on the accelerometers used?)** **(sensitivity analysis)**

| **Regression terms** | **Estimate** | **95% CI** | **p** |
| --- | --- | --- | --- |
| **Model of moderating effects of accelerometer comparability with total MVPA and ST** |  |  |  |
| MVPA:Accelerometer comparability (interaction term) | 0.006 | (-0.002, 0.013) | 0.145 |
| ST:Accelerometer comparability (interaction term) | -0.002 | (0.0003, 0.004) | 0.022* |
| **Model of moderating effects of accelerometer comparability with total ST and main effect of total MVPA** |  |  |  |
| MVPA (main effect) | -0.003 | (-0.005, -0.0008) | 0.006** |
| ST:Accelerometer comparability (interaction term) | -0.003 | (-0.004, -0.001) | <0.001*** |
| ***Accelerometer-comparability-specific effects of total ST*** |  |  |  |
| ST in those with non-comparable accelerometer | 0.002 | (-0.00002, 0.003) | 0.053 |
| ST in those with comparable accelerometer | -0.001 | (-0.002, -0.0005) | <0.001*** |

**Notes:** Model adjusted for adolescent sex, age, city, area-level walkability and SES, valid days of accelerometer wear, average wear time per day and accelerometer comparability; MVPA, moderate-to-vigorous physical activity time; ST, sedentary time; * = p<0.05; ** = p<0.01; *** = p<0.001

**Conclusion:** As when using the WHO BMI z-score as the outcome, the moderating effect of accelerometer comparability on the MVPA CDC z=score association was not significant, while that related to the ST-BMI association was significant. Participants with non-comparable accelerometers showed a positive association, while those with comparable accelerometers showed a negative association

**4a. City specific associations of total MVPA with adolescent WHO BMI z-score**

Moderating effect of city.

Note: no moderating effect of sex was found

Total MVPA and ST main effects model: AIC = 14275.79

Model with total MVPA by city and total ST by city interactions: AIC = 14256.2

Model with total ST by city interaction: AIC = 14259.95

Model with total MVPA by city interaction: AIC = 14254.95 (best fitting model)

| **Regression term** | **b** | **95%CIs** | **p** |
| --- | --- | --- | --- |
| **Model of moderating effects of city with total MVPA and main effect of total ST** |  |  |  |
| ST (main effect) | -0.001 | (-0.001, -0.001) | <0.001*** |
| MVPA (ref: Seattle, USA) | -0.007 | (-0.013, -0.001) | 0.048* |
| Interaction terms: |  |  |  |
| MVPA:Baltimore, USA | -0.003 | (-0.011, 0.006) | 0.548 |
| MVPA:Gombe, NGA | -0.008 | (-0.016, 0.000) | 0.059. |
| MVPA:Ghent, BEL | 0.001 | (-0.012, 0.013) | 0.932 |
| MVPA:Valencia, ESP | 0.011 | (0.002, 0.020) | 0.014* |
| MVPA:Porto region, PRT | 0.013 | (0.001, 0.025) | 0.030* |
| MVPA:Olomouc, CZE | 0.004 | (-0.014, 0.021) | 0.683 |
| MVPA:Hradec Králové, CZE | 0.003 | (-0.029, 0.034) | 0.870 |
| MVPA:Odense, DNK | -0.002 | (-0.015, 0.011) | 0.772 |
| MVPA:Curitiba, BRA | 0.002 | (-0.006, 0.010) | 0.934 |
| MVPA:Kuala Lumpur, MYS | 0.017 | (-0.006, 0.028) | 0.002** |
| MVPA:Melbourne, AUS | 0.005 | (-0.005, 0.016) | 0.331 |
| MVPA:Auckland, NZL | 0.006 | (-0.003, 0.014) | 0.204 |
| MVPA:Wellington, NZL | -0.0001 | (-0.011, 0.011) | 0.980 |
| MVPA:Hong Kong, CHN | 0.009 | (-0.00005, 0.017) | 0.051. |
| MVPA:Dhaka, BGD | 0.006 | (-0.006, 0.018) | 0.327 |
| MVPA:Chennai, IND | -0.007 | (-0.018, 0.002) | 0.136 |
| MVPA:Haifa, ISR | 0.005 | (-0.006, 0.016) | 0.364 |
| ***City-specific effects of MVPA*** |  |  |  |
| MVPA in Seattle, USA | -0.007 | (-0.0132, -0.0005) | 0.048* |
| MVPA in Baltimore, USA | -0.009 | (-0.015, -0.003) | 0.003** |
| MVPA in Gombe, NGA | -0.015 | (-0.020, -0.009) | <0.001*** |
| MVPA in Ghent, BEL | -0.006 | (-0.017, 0.004) | 0.251 |
| MVPA in Valencia, ESP | 0.005 | (-0.002, 0.011) | 0.159 |
| MVPA in Porto region, PRT | 0.007 | (-0.004, 0.017) | 0.203 |
| MVPA in Olomouc, CZE | -0.003 | (-0.019, 0.014) | 0.727 |
| MVPA in Hradec Králové, CZE | -0.004 | (-0.035, 0.027) | 0.796 |
| MVPA in Odense, DNK | -0.009 | (-0.020, 0.003) | 0.137 |
| MVPA in Curitiba, BRA | -0.004 | (-0.010, 0.0008) | 0.010 |
| MVPA in Kuala Lumpur, MYS | 0.010 | (0.001, 0.019) | 0.027* |
| MVPA in Melbourne, AUS | -0.001 | (-0.010, 0.007) | 0.728 |
| MVPA in Auckland, NZL | -0.001 | (-0.007, 0.005) | 0.751 |
| MVPA in Wellington, NZL | -0.007 | (-0.016, 0.002) | 0.129 |
| MVPA in Hong Kong, CHN | 0.002 | (-0.004, 0.0078) | 0.525 |
| MVPA in Dhaka, BGD | -0.0005 | (-0.011, 0.010) | 0.925 |
| MVPA in Chennai, IND | -0.014 | (-0.014, -0.023) | <0.001*** |
| MVPA in Haifa, ISR | -0.002 | (-0.010, 0.007) | 0.716 |

**Notes:** Model adjusted for adolescent sex, age, city, area-level walkability and SES, valid days of accelerometer wear, average wear time per day and accelerometer comparability. Abbreviations: AUS Australia; BGD Bangladesh; BEL Belgium; BRA Brazil; CZE Czechia; DNK Denmark; CHN China; IND India; ISR Israel; MYS Malaysia; NZL New Zealand; NGA Nigeria; PRT Portugal; ESP Spain; USA United States of America; MVPA = moderate to vigorous physical activity time; MVPA, moderate-to-vigorous physical activity; ST, sedentary time; * = p<0.05; ** = p<0.01; *** = p<0.001

**Conclusion:** There was a significant moderating effect of city on the association between MVPA and BMI. There was insufficient support for a moderating effect of city on the association between ST and BMI. This overall association was negative.

**4b: City specific associations of total MVPA with adolescent CDC BMI z-score (sensitivity analysis)**

| **Regression term** | **b** | **95%CIs** | **p** |
| --- | --- | --- | --- |
| **Model of moderating effects of city with total MVPA and main effect of total ST** |  |  |  |
| ST (main effect) | -0.0009 | (-0.002, -0.0003) | 0.006** |
| MVPA (ref: Seattle USA) | -0.005 | (-0.011, 0.0007) | 0.080. |
| Interaction terms: |  |  |  |
| MVPA:Baltimore, USA | -0.002 | (-0.010, 0.006) | 0.580 |
| MVPA:Gombe, NGA | -0.009 | (-0.016, -0.0004) | 0.040* |
| MVPA:Ghent, BEL | -0.001 | (-0.012, 0.010) | 0.866 |
| MVPA:Valencia, ESP | 0.011 | (0.003, 0.019) | 0.008** |
| MVPA:Porto region, PRT | 0.012 | (0.0006, 0.023) | 0.039* |
| MVPA:Olomouc, CZE | 0.003 | (-0.013, 0.020) | 0.689 |
| MVPA:Hradec Králové, CZE | 0.002 | (-0.027, 0.030) | 0.909 |
| MVPA:Odense, DNK | -0.002 | (-0.014, 0.010) | 0.725 |
| MVPA:Curitiba, BRA | 0.002 | (-0.006, 0.010) | 0.612 |
| MVPA:Kuala Lumpur, MYS | 0.015 | (0.005, 0.010) | 0.004** |
| MVPA:Melbourne, AUS | 0.004 | (-0.005, 0.014) | 0.367 |
| MVPA:Auckland, NZL | 0.005 | (-0.003, 0.013) | 0.210 |
| MVPA:Wellington, NZL | -0.00005 | (-0.010, 0.010) | 0.966 |
| MVPA:Hong Kong, CHN | 0.008 | (-0.0004, 0.015) | 0.063. |
| MVPA:Dhaka, BGD | 0.005 | (-0.006, 0.016) | 0.383 |
| MVPA:Chennai, IND | -0.007 | (-0.016, 0.0024) | 0.144 |
| MVPA:Haifa, ISR | 0.005 | (-0.005, 0.015) | 0.310 |
| ***City-specific effects of MVPA*** |  |  |  |
| MVPA in Seattle, USA | -0.005 | (-0.002, -0.0003) | 0.080. |
| MVPA in Baltimore, USA | -0.008 | (-0.011, 0.0007) | 0.008* |
| MVPA in Gombe, NGA | 0.013 | (-0.018, -0.008) | <0.001 |
| MVPA in Ghent, BEL | -0.006 | (-0.016, 0.003) | 0.194 |
| MVPA in Valencia, ESP | 0.006 | (-0.0002, 0.011) | 0.057. |
| MVPA in Porto region, PRT | 0.006 | (-0.003, 0.016) | 0.192 |
| MVPA in Olomouc, CZE | -0.002 | (-0.017, 0.013) | 0.790 |
| MVPA in Hradec Králové, CZE | -0.004 | (-0.032, 0.024) | 0.795 |
| MVPA in Odense, DNK | -0.008 | (-0.018, 0.003) | 0.155 |
| MVPA in Curitiba, BRA | -0.003 | (-0.008, 0.001) | 0.162 |
| MVPA in Kuala Lumpur, MYS | 0.009 | (0.001, 0.0175) | 0.027* |
| MVPA in Melbourne, AUS | -0.001 | (-0.009, 0.007) | 0.800 |
| MVPA in Auckland, NZL | -0.0003 | (-0.006, 0.005) | 0.926 |
| MVPA in Wellington, NZL | -0.005 | (-0.014, 0.003) | 0.187 |
| MVPA in Hong Kong, CHN | 0.002 | (-0.003, 0.008) | 0.449 |
| MVPA in Dhaka, BGD | -0.0004 | (-0.010, 0.009) | 0.939 |
| MVPA in Chennai, IND | -0.012 | (-0.020, -0.005) | 0.001** |
| MVPA in Haifa, ISR | -0.0003 | (-0.008, 0.008) | 0.951 |

**Notes:** Model adjusted for adolescent sex, age, city, area-level walkability and SES, valid days of accelerometer wear, average wear time per day and accelerometer comparability. Abbreviations: AUS Australia; BGD Bangladesh; BEL Belgium; BRA Brazil; CZE Czechia; DNK Denmark; CHN China; IND India; ISR Israel; MYS Malaysia; NZL New Zealand; NGA Nigeria; PRT Portugal; ESP Spain; USA United States of America; MVPA, moderate to vigorous physical activity time; ST, sedentary time; * = p<0.05; ** = p<0.01

**Conclusion:** As in the analysis with WHO BMI z-score as an outcome, city was a moderator of the associations between MVPA and CDC BMI z-score. However, it did not moderate the associations between ST and CDC BMI z-score.

**5. Accelerometer comparability as a moderator of the city by total MVPA & ST interaction effects on adolescent WHO BMI z-score (i.e, do the city by total MVPA/ST interaction effects depend on the accelerometers used?)**

**Number of Participants with comparable and non-comparable accelerometers by city**

| **City** | **not comparable** | **comparable** |
| --- | --- | --- |
| Seattle, USA | 88 | 317 |
| Baltimore, USA | 2 | 436 |
| Gombe, NGA | 0 | 245 |
| Ghent, BEL | 0 | 224 |
| Valencia, ESP | 0 | 373 |
| Porto region, PRT | 0 | 143 |
| Olomouc, CZE | 0 | 56 |
| Hradec Králové, CZE | 0 | 49 |
| Odense, DNK | 126 | 0 |
| Curitiba, BRA | 0 | 149 |
| Kuala Lumpur, MYS | 0 | 325 |
| Melbourne, AUS | 0 | 372 |
| Auckland, NZL | 0 | 340 |
| Wellington, NZL | 0 | 160 |
| Hong Kong, CHN | 0 | 549 |
| Dhaka, BGD | 0 | 90 |
| Chennai, IND | 154 | 161 |
| Haifa, ISR | 0 | 223 |

***Notes:*** *We were not able to examine the effect of accelerometer comparability on the interaction of MVPA and city on BMI because city and accelerometer comparability were collinear, as can be seen in the table above.*

Abbreviations: AUS Australia; BGD Bangladesh; BEL Belgium; BRA Brazil; CZE Czechia; DNK Denmark; CHN China; IND India; ISR Israel; MYS Malaysia; NZL New Zealand; NGA Nigeria; PRT Portugal; ESP Spain; USA United States of America; ST = sedentary time; MVPA = moderate-to-vigorous physical activity

**6a. Final model - accelerometer comparability as a moderator of associations between total ST & adolescent WHO BMI z-score in the model with city-specific effects of total MVPA**

Model with total MVPA by city interaction: AIC = 14254.95

Model with total MVPA by city and total ST by Accelerometer comparability interactions: AIC = 14249.38

Total MVPA by city interaction term: F-ratio (17, 4339) = 2.943; *p*<.001

Total ST by accelerometer interaction term: F-ratio (1, 4339) = 7.506; *p* = .006

|  | **B** | **95%CIs** | **p** |
| --- | --- | --- | --- |
| **Model of moderating effects of city with total MVPA and accelerometer comparability with total ST** |  |  |  |
| MVPA (ref: Seattle, USA) | -0.006 | (-0.012, 0.001) | 0.086. |
| Interaction terms: |  |  |  |
| MVPA:Baltimore, USA | -0.004 | (-0.012, 0.005) | 0.398 |
| MVPA:Gombe, NGA | -0.009 | (-0.017, -0.0007) | 0.034* |
| MVPA:Ghent, BEL | -0.0006 | (-0.013, 0.012) | 0.926 |
| MVPA:Valencia, ESP | 0.100 | (0.010, 0.019) | 0.026* |
| MVPA:Porto region, PRT | 0.012 | (0.0002, 0.024) | 0.045* |
| MVPA:Olomouc, CZE | 0.003 | (-0.015, 0.021) | 0.756 |
| MVPA:Hradec Králové, CZE | 0.002 | (-0.029, 0.033) | 0.893 |
| MVPA:Odense, DNK | 0.002 | (-0.011, 0.015) | 0.739 |
| MVPA:Curitiba, BRA | 0.001 | (-0.007, 0.009) | 0.773 |
| MVPA:Kuala Lumpur, MYS | 0.016 | (0.005, 0.026) | 0.005** |
| MVPA:Melbourne, AUS | 0.004 | (-0.006, 0.015) | 0.438 |
| MVPA:Auckland, NZL | 0.005 | (-0.004, 0.013) | 0.309 |
| MVPA:Wellington, NZL | -0.001 | (-0.012, 0.010) | 0.842 |
| MVPA:Hong Kong, CHN | 0.008 | (-0.001, 0.016) | 0.087. |
| MVPA:Dhaka, BGD | 0.005 | (-0.007, 0.017) | 0.405 |
| MVPA:Chennai, IND | -0.006 | (-0.016, 0.004) | 0.263 |
| MVPA:Haifa, ISR | 0.004 | (-0.007, 0.015) | 0.469 |
|  |  |  |  |
| ST (ref: non-comparable accel) | 0.001 | (-0.0007, 0.003) | 0.233 |
| ST:Accelerometer comparability (interaction term) | -0.003 | (-0.004, -0.0007) | 0.006** |
|  |  |  |  |
| ***City-specific effects of MVPA*** |  |  |  |
| MVPA in Seattle, USA | -0.006 | (-0.012, 0.0008) | 0.086 |
| MVPA in Baltimore, USA | -0.010 | (-0.016, -0.003 | 0.002** |
| MVPA in Gombe, NGA | -0.015 | (-0.020, -0.009) | <0.001*** |
| MVPA in Ghent, BEL | -0.006 | (-0.017, 0.004) | 0.233 |
| MVPA in Valencia, ESP | 0.004 | (-0.002, 0.011) | 0.177 |
| MVPA in Porto region, PRT | 0.007 | (-0.004, 0.017) | 0.212 |
| MVPA in Olomouc, CZE | -0.003 | (-0.019, 0.014) | 0.725 |
| MVPA in Hradec Králové, CZE | -0.004 | (-0.034, 0.027) | 0.815 |
| MVPA in Odense, DNK | -0.004 | (-0.015, 0.008) | 0.558 |
| MVPA in Curitiba, BRA | -0.005 | (-0.010, 0.0007) | 0.088. |
| MVPA in Kuala Lumpur, MYS | 0.010 | (0.001, 0.019) | 0.030* |
| MVPA in Melbourne, AUS | -0.002 | (-0.010, 0.007) | 0.699 |
| MVPA in Auckland, NZL | -0.001 | (-0.007, 0.005) | 0.691 |
| MVPA in Wellington, NZL | -0.007 | (-0.016, 0.002) | 0.123 |
| MVPA in Hong Kong, CHN | 0.0018 | (-0.004, 0.008) | 0.558 |
| MVPA in Dhaka, BGD | -0.0005 | (-0.011, 0.010) | 0.918 |
| MVPA in Chennai, IND | -0.012 | (-0.020, -0.003) | 0.007** |
| MVPA in Haifa, ISR | -0.002 | (-0.011, 0.007) | 0.693 |
| ***Accelerometer-comparability-specific effects of total ST*** |  |  |  |
| ST in those with non-comparable accelerometer | 0.001 | (-0.0007, 0.003) | 0.233 |
| ST in those with comparable accelerometer | -0.001 | (-0.002, -0.0007) | <0.001*** |

**Notes:** Model adjusted for adolescent sex, age, city, area-level walkability and SES, valid days of accelerometer wear, average wear time per day and accelerometer comparability. Abbreviations: AUS Australia; BGD Bangladesh; BEL Belgium; BRA Brazil; CZE Czechia; DNK Denmark; CHN China; IND India; ISR Israel; MYS Malaysia; NZL New Zealand; NGA Nigeria; PRT Portugal; ESP Spain; USA United States of America; ST = sedentary time; MVPA = moderate-to-vigorous physical activity; * = p<0.05; ** = p<0.01; *** = p<0.001

**Conclusion:** Accelerometry comparability did not determine the effect of MVPA on WHO BMI z-score, however, it moderated the effect of ST on WHO BMI z-score. For participants who did not have comparable accelerometers, the association between ST & WHO BMI z-score was not significant, but it tended to be positive. In contrast, for participants who wore comparable accelerometers, the association between ST & WHO BMI z-score was negative.

**6b. Final model - accelerometer comparability as a moderator of associations between total ST & adolescent CDC BMI z-score in the model with city-specific effects of total MVPA** **(sensitivity analysis)**

|  | **b** | **95%CIs** | **p** |
| --- | --- | --- | --- |
| **Model of moderating effects of city with total MVPA and accelerometer comparability with total ST** |  |  |  |
| MVPA (ref: Seattle, USA) | -0.005 | (-0.011, 0.001) | 0.137 |
| Interaction terms: |  |  |  |
| MVPA:Baltimore, USA | -0.003 | (-0.011, 0.005) | 0.426 |
| MVPA:Gombe, NGA | -0.009 | (-0.017, -0.001) | 0.023* |
| MVPA:Ghent, BEL | -0.002 | (-0.002, 0.009) | 0.730 |
| MVPA:Valencia, ESP | 0.010 | (0.002, 0.018) | 0.016* |
| MVPA:Porto region, PRT | 0.011 | (-0.0003, 0.022) | 0.057. |
| MVPA:Olomouc, CZE | 0.003 | (-0.014, 0.019) | 0.761 |
| MVPA:Hradec Králové, CZE | 0.001 | (-0.028, 0.030) | 0.933 |
| MVPA:Odense, DNK | 0.002 | (-0.011, 0.014) | 0.792 |
| MVPA:Curitiba, BRA | 0.001 | (-0.007, 0.009) | 0.791 |
| MVPA:Kuala Lumpur, MYS | 0.014 | (0.004, 0.024) | 0.007** |
| MVPA:Melbourne, AUS | 0.003 | (-0.006, 0.013) | 0.479 |
| MVPA:Auckland, NZL | 0.004 | (-0.004, 0.012) | 0.316 |
| MVPA:Wellington, NZL | -0.0009 | (-0.011, 0.009) | 0.860 |
| MVPA:Hong Kong, CHN | 0.007 | (-0.001, 0.015) | 0.104 |
| MVPA:Dhaka, BGD | 0.004 | (-0.007, 0.016) | 0.467 |
| MVPA:Chennai, IND | -0.005 | (-0.015, 0.004) | 0.274 |
| MVPA:Haifa, ISR | 0.004 | (-0.006, 0.014) | 0.404 |
|  |  |  |  |
| ST (ref: non-comparable accel) |  |  |  |
| ST:Accelerometer comparability (interaction term) | -0.002 | (-0.004, -0.0006) | 0.007** |
|  |  |  |  |
| ***City-specific effects of total MVPA*** |  |  |  |
| MVPA in Seattle USA | -0.005 | (-0.011, 0.001) | 0.137 |
| MVPA in Baltimore USA | -0.008 | (-0.014, -0.002) | 0.006** |
| MVPA in Gombe NGA | -0.014 | (-0.019, -0.009) | <0.001*** |
| MVPA in Ghent BEL | -0.007 | (-0.016, 0.003) | 0.180 |
| MVPA in Valencia ESP | 0.005 | (-0.0004, 0.011) | 0.065. |
| MVPA in Porto region PRT | 0.006 | (-0.003, 0.016) | 0.201 |
| MVPA in Olomouc CZE | -0.002 | (-0.017, 0.013) | 0.788 |
| MVPA in Hradec Králové CZE | -0.003 | (-0.032, 0.025) | 0.814 |
| MVPA in Odense DNK | -0.003 | (-0.014, 0.008) | 0.591 |
| MVPA in Curitiba BRA | -0.004 | (-0.008, 0.001) | 0.145 |
| MVPA in Kuala Lumpur, MYS | 0.009 | (0.0009, 0.017) | 0.030* |
| MVPA in Melbourne AUS | -0.001 | (-0.009, 0.007) | 0.770 |
| MVPA in Auckland NZL | -0.0005 | (-0.006, 0.005) | 0.864 |
| MVPA in Wellington NZL | -0.006 | (-0.014, 0.0025) | 0.180 |
| MVPA in Hong Kong CHN | 0.002 | (-0.003, 0.007) | 0.479 |
| MVPA in Dhaka BGD | -0.0004 | (-0.010, 0.009) | 0.931 |
| MVPA in Chennai IND | -0.010 | (-0.018, -0.002) | 0.013* |
| MVPA in Haifa ISR | -0.0004 | (-0.009, 0.008) | 0.927 |
| ***Accelerometer-comparability-specific effects of total ST*** |  |  |  |
| ST in those with non-comparable accelerometer | 0.001 | (-0.0005, 0.003) | 0.160 |
| ST in those with comparable accelerometer | -0.001 | (-0.002, -0.0004) | 0.002** |

**Notes:** Model adjusted for adolescent sex, age, city, area-level walkability and SES, valid days of accelerometer wear, average wear time per day and accelerometer comparability. Abbreviations: AUS Australia; BGD Bangladesh; BEL Belgium; BRA Brazil; CZE Czechia; DNK Denmark; CHN China; IND India; ISR Israel; MYS Malaysia; NZL New Zealand; NGA Nigeria; PRT Portugal; ESP Spain; USA United States of America; MVPA = moderate to vigorous physical activity time, ST = sedentary time, MVPA = moderate-to-vigorous physical activity; * = p<0.05; ** = p<0.01; *** = p<0.001

**Conclusion:** The findings when using CDC BMI z-score as the outcome variable did not differ from those based on WHO BMI z-scores. Namely, city moderated the effects of MVPA on CDC BMI z-scores and accelerometer comparability moderated the effects of ST on CDC BMI z-scores, with only participants with comparable accelerometers showing a significant negative association between ST and BMI.

. p= <0.1; * = p<0.05; ** = p<0.01; *** = p<0.001
