# Supplementary material for: Associations of accelerometer measured school- and non-school based physical activity and sedentary time with body mass index: IPEN Adolescent study
Source: Int J Behav Nutr Phys Act. 2022 Jul 14;19:85. doi: 10.1186/s12966-022-01324-x (PMC9284738; doi:10.1186/s12966-022-01324-x)
Supplement: Supplementary file 2 — Additional file 2. [file 12966_2022_1324_MOESM2_ESM.docx]

**Appendix 2: Associations of School & Non-School MVPA & ST with adolescent WHO SDS z_BMI (complete case analyses)**

**1a. Linear main effects of School & Non-School MVPA and ST on adolescent WHO BMI z-score (the best main effects model).**

|  | **b** | **95%CIs** | **p** |
| --- | --- | --- | --- |
| Average MVPA during School/valid days of wear | -0.004 | (-0.009, 0.001) | 0.114 |
| Non-School Average MVPA/ valid days of wear | -0.003 | (-0.006, 0.000) | 0.024* |
| Average ST during School/valid days of wear | -0.001 | (-0.003, 0.000) | 0.020* |
| Non-School Average ST/ valid days of wear | -0.001 | (-0.002, 0.000) | 0.165 |

**Notes:** Model adjusted for adolescent sex, age, city, area-level walkability and SES, valid days of accelerometer wear for school and non-school time, average wear time per day for school and non-school periods and accelerometer comparability. MVPA = moderate to vigorous physical activity time; ST, sedentary time; * = p<0.05

**Conclusion:** Adolescents’ WHO BMI z-scores were not significantly associated with MVPA during school time but were negatively associated with MVPA during non-school time. In contrast, WHO BMI z-scores were negatively associated with ST during school time but not significantly associated with ST during non-school time.

**1b. Linear main effects of School & Non-School MVPA and ST on adolescent CDC BMI z-score (sensitivity analysis)**

|  | **b** | **95%CIs** | **p** |
| --- | --- | --- | --- |
| Average MVPA during School/valid days of wear | -0.003 | (-0.008, 0.001) | 0.153 |
| Non-School Average MVPA/ valid days of wear | -0.003 | (-0.005, 0.0001) | 0.063. |
| Average ST during School/valid days of wear | -0.001 | (-0.003, -0.00002) | 0.047* |
| Non-School Average ST/ valid days of wear | -0.0006 | (-0.002, 0.0005) | 0.268 |

**Notes:** Model adjusted for adolescent sex, age, city, area-level walkability and SES, valid days of accelerometer wear for school and non-school time, average wear time per day for school and non-school periods and accelerometer comparability. MVPA, moderate-to-vigorous physical activity time; ST, sedentary time; . = p<0.1, * = p<0.05

**Conclusion:** As with WHO BMI z-score, CDC BMI z-score was not associated with MVPA during school time, but only marginally negatively associated with MVPA during non-school time. Also, as with WHO BMI z-score, CDC BMI z-score was negatively associated with ST during school time, but not associated with ST during non-school time. Thus, the findings for WHO BMI and CDC BMI z-scores were very similar.

**2a. Accelerometer comparability as a moderator of the associations of School & Non-School MVPA and ST with adolescent WHO BMI z-score (i.e., do the main effects depend on the accelerometers used?)**

| **Regression terms** | **Estimate** | **95% CI** | **p** |
| --- | --- | --- | --- |
| **Model of moderating effects of accelerometer comparability with School and Non-School MVPA and ST** |  |  |  |
| MVPA School:Accelerometer comparability (interaction term) | 0.012 | (-0.003, 0.028) | 0.105 |
| MVPA Non-School:Accelerometer comparability (interaction term) | 0.004 | (-0.006, 0.015) | 0.431 |
| ST School:Accelerometer comparability (interaction term) | -0.0004 | (-0.004, 0.003) | 0.832 |
| ST Non-School: Accelerometer comparability (interaction term) | -0.002 | (-0.004, -0.0005) | 0.014* |
| **Model of moderating effects of accelerometer comparability on Non-School ST and main effects of School ST and MVPA measures** |  |  |  |
| MVPA School (main effect) | -0.004 | (-0.009, 0.001) | 0.123 |
| MVPA Non-School (main effect) | -0.003 | (-0.006, -0.0004) | 0.027* |
| ST School (main effect) | -0.002 | (-0.003, -0.0003) | 0.021* |
| ST Non-School: Accelerometer comparability (interaction term) | -0.003 | (-0.005, -0.001) | 0.001** |
| ***Accelerometer-comparability-specific effects of Non-School ST*** |  |  |  |
| ST Non-School in those with non-comparable accelerometer | 0.002 | (-0.0002, 0.004) | 0.074. |
| ST Non-School in those with comparable accelerometer | -0.001 | (-0.011, 0.013) | 0.075. |

**Notes:** Model adjusted for adolescent sex, age, city, area-level walkability and SES, valid days of accelerometer wear for school and non-school time, average wear time per day for school and non-school periods and accelerometer comparability. MVPA, moderate to vigorous physical activity time; ST, sedentary time. . = p<0.1, * = p<0.05, ** = p<0.01

**Conclusion:** Accelerometry comparability did not determine the effect of School MVPA, Non-School MVPA or School ST on adolescent WHO BMI z-score. However, there was a significant difference in the effect of Non-School ST on adolescent WHO BMI z-score between those participants who had comparable or non-comparable accelerometers. While the associations of Non-School ST with WHO BMI z-score in those with non-comparable accelerometers tended to be positive, the reverse was observed in those who wore comparable accelerometers.

**3b. Accelerometer comparability as a moderator of the associations of School & Non-School MVPA and ST with adolescent CDC BMI z-score (i.e., do the main effects depend on the accelerometers used?)** **(sensitivity analysis)**

| **Regression terms** | **Estimate** | **95% CI** | **p** |
| --- | --- | --- | --- |
| **Model of moderating effects of accelerometer comparability with School and Non-School MVPA and ST** |  |  |  |
| MVPA School:Accelerometer comparability (interaction term) | 0.012 | (-0.002, 0.025) | 0.105 |
| MVPA Non-School:Accelerometer comparability (interaction term) | 0.004 | (-0.006, 0.013) | 0.473 |
| ST School:Accelerometer comparability (interaction term) | -0.0007 | (-0.004, 0.002) | 0.672 |
| ST Non-School: Accelerometer comparability (interaction term) | -0.002 | (-0.004, -0.0003) | 0.022* |
| **Model of moderating effects of accelerometer comparability with Non-School ST and main effects of School ST and MVPA measures** |  |  |  |
| MVPA School (main effect) | -0.003 | (-0.007, 0.0004) | 0.079. |
| MVPA Non-School (main effect) | -0.003 | (-0.005, -0.0005) | 0.018* |
| ST School (main effect) | -0.001 | (-0.002, -0.0006) | <0.001*** |
| ST Non-School:Accelerometer comparability (interaction term) | -0.002 | (-0.004, -0.0009) | 0.002** |
| ***Accelerometer-comparability-specific effects of Non-School ST*** |  |  |  |
| ST Non-School in those with non-comparable accelerometer | 0.001 | (-0.0002, 0.003) | 0.081. |
| ST Non-School in those with comparable accelerometer | -0.001 | (-0.002, -0.0003) | 0.003** |

**Notes:** Model adjusted for adolescent sex, age, city, area-level walkability and SES, valid days of accelerometer wear for school and non-school time, average wear time per day for school and non-school periods and accelerometer comparability. MVPA, moderate to vigorous physical activity time; ST, sedentary time. = p<0.1, * = p<0.05, ** = p<0.01, *** = p<0.001

**Conclusion:** As for WHO BMI z-score, accelerometry comparability did not determine the effect of School MVPA, Non-School MVPA or School ST on CDC BMI z-score. The associations of WHO BMI z-score & CDC BMI z-score with Non-School ST in those with non-comparable accelerometers were similar (weakly positive). For those with comparable accelerometers, the association between CDC BMI z-score and Non-School ST was significantly negative, while for WHO BMI z-score the same association was negative and approached significance (p=0.075). Overall, the moderating effect of accelerometer comparability on the associations of School & Non-School MVPA and ST with WHO BMI z-score and CDC BMI z-score were very similar.

**4. City-specific associations of School & Non-School MVPA & ST with WHO BMI z-score**

Note: no moderating effect of sex was found

City was a significant moderator of Non-School MVPA associations with adolescent WHO BMI z-score.

Non-School and School MVPA and ST main effect model: AIC = 14273.45

Non-School and School MVPA and ST by city interactions: AIC = 14289.66

Non-School MVPA/ST and School MVPA by city interactions: AIC = 14278.19

Non-School MVPA and ST by city interactions: AIC = 14257.67

Non-School MVPA by city interactions: AIC = 14255.15 (best fitting model)

| **Regression terms** | **b** | **95% CI** | **p** |
| --- | --- | --- | --- |
| **Model of moderating effects of city with Non-School MVPA and main effects of School MVPA and School and Non-School ST** |  |  |  |
| MVPA School | -0.004 | (-0.008, 0.001) | 0.144 |
| ST School | -0.002 | (-0.003, -0.0004) | 0.014* |
| MVPA Non-School (Seattle, USA) | -0.008 | (-0.016, 0.0007) | 0.073. |
| ST Non-School | -0.001 | (-0.002, 0.0005) | 0.249 |
| MVPA Non-School:Baltimore, USA | -0.003 | (-0.013, 0.008) | 0.604 |
| MVPA Non-School:Gombe, NGA | -0.009 | (-0.019, 0.002) | 0.098. |
| MVPA Non-School:Ghent, BEL | 0.009 | (-0.007, 0.025) | 0.268 |
| MVPA Non-School:Valencia, ESP | 0.014 | (0.003, 0.024) | 0.011* |
| MVPA Non-School:Porto region, PRT | 0.016 | (0.001, 0.031) | 0.033* |
| MVPA Non-School:Olomouc, CZE | 0.004 | (-0.015, 0.023) | 0.661 |
| MVPA Non-School:Hradec Králové, CZE | 0.004 | (-0.029, 0.036) | 0.834 |
| MVPA Non-School:Odense, DNK | 0.002 | (-0.015, 0.019) | 0.860 |
| MVPA Non-School:Curitiba, BRA | 0.005 | (-0.005, 0.015) | 0.339 |
| MVPA Non-School:Kuala Lumpur, MYS | 0.022 | (0.008, 0.036) | 0.002** |
| MVPA Non-School:Melbourne, AUS | 0.005 | (-0.009, 0.019) | 0.479 |
| MVPA Non-School:Auckland, NZL | 0.007 | (-0.004, 0.018) | 0.225 |
| MVPA Non-School:Wellington, NZL | -0.001 | (-0.014, 0.012) | 0.854 |
| MVPA Non-School:Hong Kong, CHN | 0.011 | (-0.00005, 0.022) | 0.051. |
| MVPA Non-School:Dhaka, BGD | 0.007 | (-0.008, 0.022) | 0.384 |
| MVPA Non-School:Chennai, IND | -0.009 | (-0.022, 0.004) | 0.183 |
| MVPA Non-School:Haifa, ISR | 0.008 | (-0.005, 0.021) | 0.239 |
|  |  |  |  |
| **City-specific effects on Non-School MVPA** |  |  |  |
| MVPA Non-School:Seattle, USA | -0.008 | (-0.016, 0.0007) | 0.073. |
| MVPA Non-School:Baltimore, USA | -0.010 | (-0.018, -0.003) | 0.007** |
| MVPA Non-School:Gombe, NGA | -0.016 | (-0.023, -0.010) | <0.001*** |
| MVPA Non-School:Ghent, BEL | 0.001 | (-0.013, 0.015) | 0.842 |
| MVPA Non-School:Valencia, ESP | 0.006 | (-0.001, 0.015) | 0.094. |
| MVPA Non-School:Porto region, PRT | 0.009 | (-0.004, 0.021) | 0.185 |
| MVPA Non-School:Olomouc, CZE: | -0.003 | (-0.002, 0.014) | 0.701 |
| MVPA Non-School:Hradec Králové, CZE | -0.004 | (-0.036, 0.028) | 0.803 |
| MVPA Non-School:Odense, DNK: | -0.006 | (-0.021, 0.009) | 0.436 |
| MVPA Non-School:Curitiba, BRA | -0.003 | (-0.009, 0.0037) | 0.418 |
| MVPA Non-School:Kuala Lumpur, MYS | 0.014 | (0.003, 0.026) | 0.015* |
| MVPA Non-School:Melbourne, AUS | -0.003 | (-0.013, 0.009) | 0.652 |
| MVPA Non-School:Auckland, NZL | -0.0008 | (-0.008, 0.007) | 0.830 |
| MVPA Non-School:Wellington, NZL | -0.009 | (-0.019, 0.001) | 0.092. |
| MVPA Non-School:Hong Kong, CHN | 0.003 | (-0.005, 0.011) | 0.400 |
| MVPA Non-School:Dhaka, BGD | -0.0009 | (-0.014, 0.012) | 0.893 |
| MVPA Non-School:Chennai, IND | -0.017 | (-0.028, -0.006) | 0.003** |
| MVPA Non-School:Haifa, ISR | 0.0003 | (-0.010, 0.011) | 0.961 |

**Notes:** Model adjusted for adolescent sex, age, city, area-level walkability and SES, valid days of accelerometer wear for school and non-school time, average wear time per day for school and non-school periods and accelerometer comparability. Abbreviations: AUS Australia; BGD Bangladesh; BEL Belgium; BRA Brazil; CZE Czechia; DNK Denmark; CHN China; IND India; ISR Israel; MYS Malaysia; NZL New Zealand; NGA Nigeria; PRT Portugal; ESP Spain; USA United States of America; MVPA = moderate to vigorous physical activity time; MVPA, moderate-to-vigorous physical activity; ST, sedentary time; * = p<0.05; ** = p<0.01; *** = p<0.001

**Conclusion:** City did not moderate the associations of School MVPA and ST and Non-School ST with WHO BMI z-score. As in model 1a (see above), the main effects of MVPA School and ST Non-School on WHO BMI z-score were not significant, whereas ST School was negatively associated with WHO BMI z-score. City was a moderator or the associations between Non-School MVPA and WHO BMI z-score. Seattle, Baltimore, Gombe, Wellington and Chennai had negative while Valencia and Kuala Lumpar had positive associations of Non-School MVPA with WHO BMI z-score.

**5. City-specific associations of School & Non-School MVPA & ST with CDC BMI z-score**

Note: no moderating effect of sex was found

City was a significant moderator of Non-School MVPA associations with adolescent CDC BMI z-score.

Non-School and School MVPA and ST main effect model: AIC = 13581.87

Non-School and School MVPA and ST by city interactions: AIC = 13598.11

Non-School MVPA/ST and School MVPA by city interactions: AIC = 13585.87

Non-School MVPA and ST by city interactions: AIC = 13565.47

Non-School MVPA by city interactions: AIC = 13562.95 (best fitting model)

| **Regression terms** | **b** | **95% CI** | **p** |
| --- | --- | --- | --- |
| **Model of moderating effects of city with Non-School MVPA and main effects of School MVPA and School and Non-School ST** |  |  |  |
| MVPA School | -0.003 | (-0.008, 0.002) | 0.202 |
| ST School | -0.001 | (-0.003, -0.0001) | 0.034* |
| MVPA Non-School (Seattle, USA) | -0.006 | (-0.014, 0.001) | 0.114 |
| ST Non-School | -0.0005 | (-0.002, 0.0006) | 0.394 |
| MVPA Non-School:Baltimore, USA | -0.003 | (-0.012, 0.007) | 0.618 |
| MVPA Non-School:Gombe, NGA | -0.009 | (-0.018, 0.0005) | 0.064. |
| MVPA Non-School:Ghent, BEL | 0.007 | (-0.008, 0.021) | 0.373 |
| MVPA Non-School:Valencia, ESP | 0.013 | (0.004, 0.023) | 0.008** |
| MVPA Non-School:Porto region, PRT | 0.014 | (0.0005, 0.028) | 0.042* |
| MVPA Non-School:Olomouc, CZE | 0.003 | (-0.014, 0.021) | 0.702 |
| MVPA Non-School:Hradec Králové, CZE | 0.003 | (-0.028, 0.033) | 0.871 |
| MVPA Non-School:Odense, DNK | 0.001 | (-0.014, 0.017) | 0.877 |
| MVPA Non-School:Curitiba, BRA | 0.004 | (-0.005, 0.014) | 0.368 |
| MVPA Non-School:Kuala Lumpur, MYS | 0.019 | (0.007, 0.032) | 0.003** |
| MVPA Non-School:Melbourne, AUS | 0.004 | (-0.008, 0.017) | 0.513 |
| MVPA Non-School:Auckland, NZL | 0.006 | (-0.004, 0.016) | 0.252 |
| MVPA Non-School:Wellington, NZL | -0.001 | (-0.013, 0.011) | 0.872 |
| MVPA Non-School:Hong Kong, CHN | 0.009 | (-0.0008, 0.019) | 0.071. |
| MVPA Non-School:Dhaka, BGD | 0.005 | (-0.009, 0.019) | 0.458 |
| MVPA Non-School:Chennai, IND | -0.008 | (-0.020, 0.004) | 0.202 |
| MVPA Non-School:Haifa, ISR | 0.007 | (-0.005, 0.020) | 0.223 |
|  |  |  |  |
| **City-specific effects on Non-School MVPA** |  |  |  |
| MVPA in Seattle, USA | -0.006 | (-0.014, 0.001) | 0.114 |
| MVPA in Baltimore, USA | -0.009 | (-0.016, -0.002) | 0.015* |
| MVPA in Gombe, NGA | -0.015 | (-0.021, -0.009) | <0.001*** |
| MVPA in Ghent, BEL | 0.0005 | (-0.012, 0.013) | 0.938 |
| MVPA in Valencia, ESP | 0.007 | (0.0005, 0.014) | 0.033* |
| MVPA in Porto region, PRT | 0.008 | (-0.004, 0.020) | 0.176 |
| MVPA in Olomouc, CZE | -0.003 | (-0.018, 0.013) | 0.731 |
| MVPA in Hradec Králové, CZE | -0.004 | (-0.033, 0.026) | 0.809 |
| MVPA in Odense, DNK | -0.005 | (-0.019, 0.009) | 0.493 |
| MVPA in Curitiba, BRA | -0.002 | (-0.008, 0.004) | 0.532 |
| MVPA in Kuala Lumpur, MYS | 0.013 | (0.003, 0.024) | 0.015* |
| MVPA in Melbourne, AUS | -0.002 | (-0.012, 0.009) | 0.718 |
| MVPA in Auckland, NZL | -0.0003 | (-0.007, 0.007) | 0.935 |
| MVPA in Wellington, NZL | -0.007 | (-0.017, 0.002) | 0.138 |
| MVPA in Hong Kong, CHN | 0.003 | (-0.004, 0.010) | 0.392 |
| MVPA in Dhaka, BGD | -0.0009 | (-0.013, 0.011) | 0.882 |
| MVPA in Chennai, IND | -0.014 | (-0.024, -0.004) | 0.007** |
| MVPA in Haifa, ISR | 0.001 | (-0.008, 0.011) | 0.786 |

**Notes:** Model adjusted for adolescent sex, age, city, area-level walkability and SES, valid days of accelerometer wear for school and non-school time, average wear time per day for school and non-school periods and accelerometer comparability. Abbreviations: AUS, Australia; BGD, Bangladesh; BEL, Belgium; BRA, Brazil; CZE, Czechia; DNK, Denmark; CHN, China; IND, India; ISR, Israel; MYS, Malaysia; AUS, Australia; NZL, New Zealand; NGA, Nigeria; PRT, Portugal; ESP, Spain; USA, United States of America; MVPA, moderate-to-vigorous physical activity time; ST, sedentary time; SES, area-level socio-economic status; . = p<0.1 * = p<0.05; ** = p<0.01; *** = p<0.001

**Conclusion:** City did not moderate the associations of School MVPA and ST and Non-School ST with CDC BMI z-score. The main effects of School MVPA and Non-School ST on CDC BMI z-score were not significant, whereas School ST was negatively associated with CDC BMI z-score. City moderated the effects of Non-School MVPA on CDC BMI z-score. City-specific associations of Non-School MVPA with CDC BMI z-score were very similar to those observed for WHO BMI z-score.

**6a. Final model - accelerometer comparability as a moderator of associations between Non-School ST & adolescent WHO BMI z-score in the model with city-specific effects of Non-School MVPA**

Model with Non-School MVPA by city interaction: AIC = 14255.15

Model with Non-School MVPA by city and Non-School ST by Accelerometer comparability interactions: AIC = 14249.18

Non-School MVPA by city interaction term: F-ratio (17, 4339) = 2.926; *p*<.001

Non-School ST by accelerometer interaction term: F-ratio (1, 4339) = 7.886; *p* = .005

| **Regression terms** | **b** | **95% CI** | **p** |
| --- | --- | --- | --- |
| **Model of moderating effects of city with Non-School MVPA, accelerometer comparability with Non-School ST and main effects of School MVPA and ST** |  |  |  |
| School MVPA (main effect) | -0.004 | (-0.009, 0.001) | 0.153 |
| School ST (main effect) | -0.002 | (-0.003, -0.0004) | 0.015* |
| Non-School MVPA (ref: Seattle, USA) | -0.007 | (-0.015, 0.002) | 0.114 |
| Interaction terms: |  |  |  |
| MVPA:Baltimore, USA | -0.004 | (-0.015, 0.007) | 0.474 |
| MVPA:Gombe, NGA | -0.010 | (-0.020, 0.0007) | 0.067. |
| MVPA:Ghent, BEL | 0.008 | (-0.008, 0.024) | 0.318 |
| MVPA:Valencia, ESP | 0.012 | (0.002, 0.023) | 0.020* |
| MVPA:Porto region, PRT | 0.015 | (0.0003, 0.030) | 0.046* |
| MVPA:Olomouc, CZE | 0.004 | (-0.020, 0.022) | 0.714 |
| MVPA:Hradec Králové, CZE | 0.003 | (-0.030, 0.036) | 0.859 |
| MVPA:Odense, DNK | 0.005 | (-0.012, 0.022) | 0.596 |
| MVPA:Curitiba, BRA | 0.004 | (-0.006, 0.014) | 0.447 |
| MVPA:Kuala Lumpur, MYS | 0.021 | (0.007, 0.035) | 0.003** |
| MVPA:Melbourne, AUS | 0.004 | (-0.010, 0.018) | 0.574 |
| MVPA:Auckland, NZL | 0.006 | (-0.005, 0.016) | 0.314 |
| MVPA:Wellington, NZL | -0.002 | (-0.015, 0.011) | 0.729 |
| MVPA:Hong Kong, CHN | 0.010 | (-0.001, 0.021) | 0.076. |
| MVPA:Dhaka, BGD | 0.006 | (-0.009, 0.021) | 0.455 |
| MVPA:Chennai, IND | -0.008 | (-0.022, 0.005) | 0.225 |
| MVPA:Haifa, ISR | 0.007 | (-0.006, 0.020) | 0.294 |
|  |  |  |  |
| ST (ref: non-comparable accelerometer) | 0.002 | (-0.0003, 0.004) | 0.102 |
| ST:Accelerometer comparability  (interaction term) | -0.003 | (-0.004, -0.0008) | 0.005** |
|  |  |  |  |
| ***City-specific effects of* Non-School MVPA on WHO BMI z-score** |  |  |  |
| MVPA in Seattle, USA | -0.007 | (-0.015, 0.002) | 0.114 |
| MVPA in Baltimore, USA | -0.011 | (-0.018, -0.003) | 0.006** |
| MVPA in Gombe, NGA | -0.016 | (-0.023, -0.010) | <0.001*** |
| MVPA in Ghent, BEL | 0.001 | (-0.012, 0.015) | 0.842 |
| MVPA in Valencia, ESP | 0.006 | (-0.001, 0.013) | 0.108 |
| MVPA in Porto region, PRT | 0.008 | (-0.004, 0.021) | 0.191 |
| MVPA in Olomouc, CZE | -0.003 | (-0.020, 0.014) | 0.716 |
| MVPA in Hradec Králové, CZE | -0.004 | (-0.036, 0.028 | 0.820 |
| MVPA in Odense, DNK | -0.002 | (-0.017, 0.013) | 0.794 |
| MVPA in Curitiba, BRA | -0.003 | (-0.009, 0.004) | 0.397 |
| MVPA in Kuala Lumpur, MYS | 0.014 | (0.003, 0.026) | 0.016* |
| MVPA in Melbourne, AUS | -0.003 | (-0.014, 0.009) | 0.635 |
| MVPA in Auckland, NZL | -0.001 | (-0.009, 0.006) | 0.779 |
| MVPA in Wellington, NZL | -0.009 | (-0.019, 0.001) | 0.085. |
| MVPA in Hong Kong, CHN | 0.003 | (-0.005, 0.011) | 0.416 |
| MVPA in Dhaka, BGD | -0.001 | (-0.014, 0.012) | 0.885 |
| MVPA in Chennai, IND | -0.015 | (-0.026, -0.004) | 0.009** |
| MVPA in Haifa, ISR | 0.0003 | (-0.010, 0.011) | 0.956 |
| ***Accelerometer-comparability-specific effects of* Non-School ST on WHO BMI z-score** |  |  |  |
| ST in those with non-comparable accelerometers | 0.002 | (-0.0003, 0.0004) | 0.102 |
| ST in those with comparable accelerometers | -0.001 | (-0.002, 0.0003) | 0.138 |

**Notes:** Model adjusted for adolescent sex, age, city, area-level walkability and SES, valid days of accelerometer wear for school and non-school time, average wear time per day for school and non-school periods and accelerometer comparability. Abbreviations: AUS Australia; BGD Bangladesh; BEL Belgium; BRA Brazil; CZE Czechia; DNK Denmark; CHN China; IND India; ISR Israel; MYS Malaysia; NZL New Zealand; NGA Nigeria; PRT Portugal; ESP Spain; USA United States of America; = p<0.1 * = p<0.05; ** = p<0.01; *** = p<0.001

**Conclusion:** School MVPA was not significantly associated with WHO BMI z-score, while a negative association was observed with School ST. Accelerometer comparability determined the effects of Non-School ST on WHO BMI z-score. For participants who did not have comparable accelerometers, the association between Non-School ST & WHO BMI z-score was not significant, but it tended to be positive. In contrast, for participants who wore comparable accelerometers, the association between Non-School ST & WHO BMI z-score tended to be negative. With regard to city-specific associations of Non-School MVPA with WHO BMI z-score, they were negative for Baltimore, Gombe, Wellington and Chennai and positive for the Malaysian cities.

**6b. Final model - accelerometer comparability as a moderator of associations between Non-School ST & adolescent CDC BMI z-score in the model with city-specific effects of Non-School MVPA**

Model with Non-School MVPA by city interaction: AIC = 13562.95

Model with Non-School MVPA by city and Non-School ST by Accelerometer comparability interactions: AIC = 13557.74

Non-School MVPA by city interaction term: F-ratio (17, 4339) = 2.973; *p*<.001

Non-School ST by accelerometer interaction term: F-ratio (1, 4339) = 7.138; *p* = .008

| **Regression terms** | **B** | **95% CI** | **P** |
| --- | --- | --- | --- |
| **Model of moderating effects of city with Non-School MVPA, accelerometer comparability with Non-School ST and main effects of School MVPA and ST** |  |  |  |
| School MVPA (main effect) | -0.003 | (-0.008, 0.002) | 0.213 |
| School ST (main effect) | -0.001 | (-0.003, -0.0001) | 0.037* |
| Non-School MVPA (ref: Seattle, USA) | -0.005 | (-0.013, 0.002) | 0.168 |
| Interaction terms: |  |  |  |
| MVPA:Baltimore, USA | -0.003 | (-0.003, 0.005) | 0.492 |
| MVPA:Gombe, NGA | -0.010 | (-0.019, -0.0003) | 0.043* |
| MVPA:Ghent, BEL | 0.006 | (-0.009, 0.021) | 0.431 |
| MVPA:Valencia, ESP | 0.012 | (0.003, 0.022) | 0.014* |
| MVPA:Porto region, PRT | 0.013 | (-0.0003, 0.027) | 0.055. |
| MVPA:Olomouc, CZE | 0.003 | (-0.015, 0.013 | 0.753 |
| MVPA:Hradec Králové, CZE | 0.002 | (-0.028, 0.032) | 0.895 |
| MVPA:Odense, DNK | 0.004 | (-0.012, 0.020) | 0.623 |
| MVPA:Curitiba, BRA | 0.003 | (-0.006, 0.013) | 0.475 |
| MVPA:Kuala Lumpur, MYS | 0.018 | (0.006, 0.031) | 0.005** |
| MVPA:Melbourne, AUS | 0.003 | (-0.009, 0.016) | 0.606 |
| MVPA:Auckland, NZL | 0.005 | (-0.005, 0.015) | 0.343 |
| MVPA:Wellington, NZL | -0.002 | (-0.014, 0.010) | 0.753 |
| MVPA:Hong Kong, CHN | 0.008 | (-0.002, 0.019) | 0.102 |
| MVPA:Dhaka, BGD | 0.004 | (-0.009, 0.018) | 0.533 |
| MVPA:Chennai, IND | -0.007 | (-0.020, 0.005) | 0.244 |
| MVPA:Haifa, ISR | 0.007 | (-0.005 0.019) | 0.271 |
|  |  |  |  |
| ST (ref: non-comparable accelerometer) | 0.002 | (-0.0002, 0.003) | 0.090. |
| ST:Accelerometer comparability  (interaction term) | -0.002 | (-0.004, -0.0006) | 0.008** |
|  |  |  |  |
| ***City-specific effects of MVPA*** |  |  |  |
| MVPA in Seattle, USA | -0.005 | (-0.013, 0.002) | 0.168 |
| MVPA in Baltimore, USA | -0.009 | (-0.016, -0.002) | 0.013* |
| MVPA in Gombe, NGA | -0.015 | (-0.021, -0.009) | <0.001*** |
| MVPA in Ghent, BEL | 0.0005 | (-0.012, 0.013) | 0.938 |
| MVPA in Valencia, ESP | 0.007 | (0.0003, 0.013) | 0.039* |
| MVPA in Porto region, PRT | 0.008 | (-0.004, 0.020) | 0.181 |
| MVPA in Olomouc, CZE | -0.003 | (-0.018, 0.013) | 0.747 |
| MVPA in Hradec Králové, CZE | -0.003 | (-0.033, 0.026) | 0.824 |
| MVPA in Odense, DNK | -0.001 | (-0.012, 0.020) | 0.846 |
| MVPA in Curitiba, BRA | -0.002 | (-0.008, 0.004) | 0.510 |
| MVPA in Kuala Lumpur, MYS | 0.013 | (0.002, 0.024) | 0.017* |
| MVPA in Melbourne, AUS | -0.002 | (-0.013, 0.008) | 0.701 |
| MVPA in Auckland, NZL | -0.0005 | (-0.008, 0.006) | 0.885 |
| MVPA in Wellington, NZL | -0.007 | (-0.017, 0.002) | 0.129 |
| MVPA in Hong Kong, CHN | 0.003 | ( -0.004, 0.010) | 0.406 |
| MVPA in Dhaka, BGD | -0.001 | (-0.013, 0.011) | 0.875 |
| MVPA in Chennai, IND | -0.013 | (-0.023, -0.002) | 0.016* |
| MVPA in Haifa, ISR | 0.001 | (-0.008, 0.011) | 0.781 |
| ***Accelerometer-comparability-specific effects of* Non-School ST on CDC BMI z-score** |  |  |  |
| ST in those with non-comparable accelerometers | 0.002 | (-0.0002, 0.003) | 0.090 |
| ST in those with comparable accelerometers | -0.001 | (-0.002, 0.0004) | 0.247 |

**Notes:** Model adjusted for adolescent sex, age, city, area-level walkability and SES, valid days of accelerometer wear for school and non-school time, average wear time per day for school and non-school periods and accelerometer comparability. Abbreviations: AUS Australia; BGD Bangladesh; BEL Belgium; BRA Brazil; CZE Czechia; DNK Denmark; CHN China; IND India; ISR Israel; MYS Malaysia; NZL New Zealand; NGA Nigeria; PRT Portugal; ESP Spain; USA United States of America; = p<0.1 * = p<0.05; ** = p<0.01; *** = p<0.001

The opposite was observed for those with comparable accelerometers. With regard to city-specific associations of Non-School MVPA with WHO BMI z-score, they were negative for Baltimore, Gombe, Wellington and Chennai and positive for the Malaysian cities.

**Conclusion:** As for WHO BMI z-score, School MVPA was not a significant correlate of CDC BMI z-score, while School ST was negatively related to CDC BMI z-score. Accelerometer comparability determined the effect of Non-School ST on CDC BMI z-score. For participants who did not have comparable accelerometers, the association between Non-School ST and CDC BMI z-score tended to be positive (p<0.1) as was observed for WHO BMI z-score. In contrast, the associations between Non-School ST & WHO BMI z-score and Non-School ST & CDC BMI z-score for participants who wore comparable accelerometers tended to be both negative. With regard to city-specific association of Non-School MVPA with WHO BMI z-score, they were negative for Baltimore, Gombe, Wellington (p<0.1) and Chennai and positive for and the Malaysian cities. The CDC BMI z-score yielded similar city-specific associations with the exception of Wellington (weaker association) and Valencia (positive association).
