# Supplementary material for: Associations of accelerometer measured school- and non-school based physical activity and sedentary time with body mass index: IPEN Adolescent study
Source: Int J Behav Nutr Phys Act. 2022 Jul 14;19:85. doi: 10.1186/s12966-022-01324-x (PMC9284738; doi:10.1186/s12966-022-01324-x)
Supplement: Supplementary file 3 — Additional file 3. [file 12966_2022_1324_MOESM3_ESM.docx]

**Appendix 3: Associations between being overweight/obese (ITOF BMI categories: thin/normal vs. overweight/obese) and total MVPA & ST (complete cases analyses)**

**1. Associations of socio-demographic characteristics with being overweight/obese (ITOF BMI categories)**

|  | **OR** | **95%CIs** | **p** |
| --- | --- | --- | --- |
| Sex (ref: male) |  |  |  |
| Female | 0.750 | (0.641, 0.877) | <0.001*** |
| Age (range 11-19 years) | 1.025 | (0.971, 1.083) | 0.371 |
| City (ref: Seattle, USA) |  |  |  |
| Baltimore, USA | 1.230 | (0.8900, 1.701) | 0.210 |
| Gombe, NGA | 0.215 | (0.121, 0.380) | <0.001*** |
| Ghent, BEL | 0.355 | (0.204, 0.615) | <0.001*** |
| Valencia, ESP | 0.778 | (0.529, 1.145) | 0.203 |
| Porto region, PRT | 1.709 | (1.049, 2.782) | 0.031* |
| Olomouc, CZE | 0.449 | (0.148, 1.357) | 0.156 |
| Hradec Králové, CZE | 0.319 | (0.070, 1.461) | 0.141 |
| Odense, DNK | 0.322 | (0.158, 0.658) | 0.002** |
| Curitiba, BRA | 1.420 | (1.023, 1.972) | 0.036* |
| Kuala Lumpur & others, MYS | 0.942 | (0.584, 1.518) | 0.806 |
| Melbourne, AUS | 0.905 | (0.575, 1.423) | 0.666 |
| Auckland, NZL | 1.033 | (0.716, 1.490) | 0.861 |
| Wellington, NZL | 0.761 | (0.472, 1.228) | 0.263 |
| Hong Kong, CHN | 0.351 | (0.236, 0.521) | <0.001*** |
| Dhaka, BGD | 0.773 | (0.396, 1.511) | 0.452 |
| Chennai, IND | 1.005 | (0.701, 1.440) | 0.978 |
| Haifa, ISR | 0.549 | (0.344, 0.877) | 0.012* |
| Education (ref: <college) |  |  |  |
| ≥college | 0.812 | (0.688, 0.958) | 0.014* |
| Walkability (ref: low) |  |  |  |
| High | 0.928 | (0.789, 1.091) | 0.364 |
| SES (ref: low) |  |  |  |
| High | 0.842 | (0.715, 0.993) | 0.040* |

**Notes:** Abbreviations: AUS, Australia; BGD, Bangladesh; BEL, Belgium; BRA, Brazil; CZE, Czechia; DNK, Denmark; CHN, China; IND, India; ISR, Israel; MYS, Malaysia; NZL, New Zealand; NGA, Nigeria; PRT, Portugal; ESP, Spain; USA, United States of America; MVPA, moderate-to-vigorous physical activity time; ST, sedentary time; SES, area-level socio-economic status; * = p<0.05; ** = P<0.01; *** = p<0.001

**Conclusion:** Female adolescents were less likely to be overweight/obese than male adolescents. Adolescents from high socio-economic status neighbourhoods and those from a household with higher education were less likely to be overweight/obese than their counterparts. There was no significant association of weight status with adolescent age. Adolescents from two cities had significantly higher odds of being overweight/obese than the reference city (Seattle USA), while adolescents from six cities (Gombe, Ghent, Odense, Hong Kong, Haifa and Valencia) were significantly less likely than Seattle adolescents to be overweight/obese.

**MAIN EFFECTS – COMPLETE CASES**

**The best main effects model was that with linear terms for MVPA & ST.**

**2. Linear main effects of MVPA and ST on being overweight/obese (ITOF BMI categories) (the best main effects model).**

|  | **OR** | **95%CIs** | **p** |
| --- | --- | --- | --- |
| MVPA (min/day) | 0.991 | (0.986, 0.995) | <0.001*** |
| ST (min/day) | 0.997 | (0.996, 0.999) | <0.001*** |

**Notes:** Model adjusted for adolescent sex, age, city, area-level walkability and SES, valid days of accelerometer wear, average wear time per day and accelerometer comparability; MVPA, moderate-to-vigorous physical activity time; ST, sedentary time; *** = p<0.001

**Conclusion:**

Both total MVPA and ST were negatively associated with the odds of being overweight/obese in adolescents.

**3. Accelerometer comparability as a moderator of the main effects of MVPA & ST on being overweight/obese (ITOF BMI categories) (i.e, do the main effects depend on the accelerometers used?)**

| **Regression terms** | **OR** | **95% CI** | **p** |
| --- | --- | --- | --- |
| **Model of moderating effects of accelerometer comparability with total MVPA and ST** |  |  |  |
| MVPA:Accelerometer comparability (interaction term) | 1.012 | (0.992, 1.033) | 0.249 |
| ST:Accelerometer comparability (interaction term) | 0.997 | (0.993,1.002) | 0.214 |
| **Model of moderating effects of accelerometer comparability with total ST and main effect of total MVPA** |  |  |  |
| MVPA (main effect) | 0.991 | (0.986, 0.996) | <0.001*** |
| ST:Accelerometer comparability (interaction term) | 0.996 | (0.992, 0.9998) | 0.041* |
| ***Accelerometer-comparability-specific effects of total ST*** |  |  |  |
| ST in those with non-comparable accelerometer | 1.001 | (0.997, 1.005) | 0.621 |
| ST in those with comparable accelerometer | 0.997 | (0.996, 0.999) | <0.001*** |

**Notes:** Model adjusted for adolescent sex, age, city, area-level walkability and SES, valid days of accelerometer wear, average wear time per day and accelerometer comparability; MVPA, moderate-to-vigorous physical activity time; ST, sedentary time

**Conclusion:** Accelerometer comparability moderated the association of total ST with being overweight/obese but did not moderate the effect of total MVPA on being overweight/obese. Total MVPA was significantly negatively associated with the odds of being overweight/obese in all adolescents, while total ST showed a negative association only in those with comparable accelerometers.

**4. City- or sex-specific associations of total MVPA & ST with being overweight/obese (ITOF BMI categories)**

**Insufficient evidence of moderating effects of city or sex on associations between total MVPA/ST and being overweight/obese was found.**

Linear model with CITY AS A MODERATOR OF MVPA & ST: Worse than linear main effect model (AIC = 20957.48)

Linear model with CITY AS A MODERATOR OF MVPA: Worse than linear main effect model (AIC = 20851.92)

Linear model with CITY AS A MODERATOR OF ST: Worse than linear main effect model (AIC = 20827.43)

Linear model with GENDER AS A MODERATOR of MVPA & ST: Worse than linear main effect model (AIC = 20725.8)

linear model with GENDER AS A MODERATOR of MVPA: Equal to linear main effect model (AIC = 20721.94)

Linear model with GENDER AS A MODERATOR of ST: Worse than linear main effect model (AIC = 20725.02)

These models had greater or equal AICs than the linear main effects model (AIC = 20721.98).
