# Supplementary material for: Associations of accelerometer measured school- and non-school based physical activity and sedentary time with body mass index: IPEN Adolescent study
Source: Int J Behav Nutr Phys Act. 2022 Jul 14;19:85. doi: 10.1186/s12966-022-01324-x (PMC9284738; doi:10.1186/s12966-022-01324-x)
Supplement: Supplementary file 4 — Additional file 4. [file 12966_2022_1324_MOESM4_ESM.docx]

**Appendix 4: Associations between weight status (thin/normal vs. overweight/obese; ITOF BMI categories) and School & Non-School MVPA & ST (main effects – complete cases)**

**1. Linear main effects of School & Non-School MVPA and ST on being overweight/obese (ITOF BMI categories) (the best main effects model).**

|  | **OR** | **95%CIs** | **p** |
| --- | --- | --- | --- |
| MVPA during School/valid days of wear | 0.992 | (0.982, 1.003) | 0.154 |
| Non-School MVPA/ valid days of wear | 0.991 | (0.985, 0.998) | 0.007** |
| ST during School/valid days of wear | 0.996 | (0.993, 0.999) | 0.011* |
| Non-School ST/ valid days of wear | 0.998 | (0.996, 1.001) | 0.196 |

**Notes:** Model adjusted for adolescent sex, age, city, area-level walkability and SES, valid days of accelerometer wear, average wear time per day and accelerometer comparability. MVPA, moderate to vigorous physical activity time; ST, sedentary time; * = p<0.05, ** = p<0.01

**Conclusion:** Being overweight/obese was not associated with MVPA during school time, but higher levels of MVPA during non-school time were associated with lower odds of being overweight/obese. In contrast, the likelihood of being overweight/obese was negatively associated with ST during school time, but not associated with ST during non-school time.

**3. Accelerometer comparability as a moderator of the main effects of School and Non-School MVPA & ST on being overweight/obese (ITOF BMI categories) (i.e, do the main effects depend on the accelerometers used?)**

| **Regression terms** | **Estimate** | **95% CI** | **p** |
| --- | --- | --- | --- |
| **Model of moderating effects of accelerometer comparability with School and Non-School MVPA and ST** |  |  |  |
| MVPA School:Accelerometer comparability (interaction term) | 1.002 | (0.965, 1.041) | 0.907 |
| MVPA Non-School:Accelerometer comparability (interaction term) | 1.026 | (0.996, 1.056) | 0.092. |
| ST School:Accelerometer comparability (interaction term) | 1.002 | (0.995, 1.056) | 0.539 |
| ST Non-School: Accelerometer comparability (interaction term) | 0.997 | (0.993, 1.001) | 0.119 |
| **Model of moderating effects of accelerometer comparability with Non-School ST and main effects of School ST and MVPA measures** |  |  |  |
| MVPA School (main effect) | 0.992 | (0.982, 1.003) | 0.164 |
| MVPA Non-School (main effect) | 0.991 | (0.985, 0.998) | 0.007** |
| ST School (main effect) | 0.996 | (0.993, 0.999) | 0.012* |
| ST Non-School:Accelerometer comparability (interaction term) | 0.996 | (0.992, 0.9997) | 0.034* |
| ***Accelerometer-comparability-specific effects of Non-School ST*** |  |  |  |
| ST Non-School in those with non-comparable accelerometer | 1.002 | (0.998, 1.007) | 0.282 |
| ST Non-School in those with comparable accelerometer | 0.998 | (0.996, 1.000) | 0.121 |

**Notes:** Model adjusted for adolescent sex, age, city, area-level walkability and SES, valid days of accelerometer wear, average wear time per day and accelerometer comparability; MVPA, moderate to vigorous physical activity time; ST, sedentary time; . = p<0.1, * = p<0.05, ** = p<0.001

**Conclusion:** Accelerometry comparability did not determine the effect of School MVPA, School ST or Non-School ST on being overweight/obese (ITOF BMI categories). However, there were significant differences in the effect of Non-School ST on being overweight/obese between those participants who had comparable or non-comparable accelerometers, with the former tending to show a negative and the latter a positive association.

**4. City- or sex-specific associations of School & Non-School MVPA & ST with being overweight/obese (ITOF BMI categories)**

Insufficient evidence of moderating effects of city or sex on associations between School and Non-School MVPA/ST and being overweight/obese (ITOF BMI categories) was found.
