# Supplementary material for: Associations of accelerometer measured school- and non-school based physical activity and sedentary time with body mass index: IPEN Adolescent study
Source: Int J Behav Nutr Phys Act. 2022 Jul 14;19:85. doi: 10.1186/s12966-022-01324-x (PMC9284738; doi:10.1186/s12966-022-01324-x)
Supplement: Supplementary file 5 — Additional file 5. [file 12966_2022_1324_MOESM5_ESM.doc]

STROBE Statement—Checklist of items that should be included in reports of ***cross-sectional studies***

|  | Item No | Recommendation |
| --- | --- | --- |
| **Title and abstract** | 1 | (*a*) Indicate the study’s design with a commonly used term in the title or the abstract Study design was mentioned in the abstract of the paper |
|  |  | (*b*) Provide in the abstract an informative and balanced summary of what was done and what was found OK, see abstract |
| Introduction | | |
| Background/rationale | 2 | Explain the scientific background and rationale for the investigation being reported OK, see background section of the manuscript |
| Objectives | 3 | State specific objectives, including any prespecified hypotheses Objectives are clearly stated at the end of the background section. We did not have prespecified hypotheses to test in this study, so these were not included. (Page 3 of the ‘MainDocument’) |
| Methods | | |
| Study design | 4 | Present key elements of study design early in the paper In the first paragraph of the Methods section, the study design of the study is explained (page 3 of the ‘MainDocument’) |
| Setting | 5 | Describe the setting, locations, and relevant dates, including periods of recruitment, exposure, follow-up, and data collection All of this was explained in the methods section (pages 3 an 4 of the ‘MainDocument’) and we refer to the published protocol paper of IPEN Adolescent for further details (Cain et al, BMJ Open, 2021) |
| Participants | 6 | (*a*) Give the eligibility criteria, and the sources and methods of selection of participants This was explained in the methods section (Participant Recruitment, page 4 of the ‘MainDocument’). Furthermore, we refer to the published protocol paper of IPEN Adolescent for further details (Cain et al, BMJ Open, 2021) |
| Variables | 7 | Clearly define all outcomes, exposures, predictors, potential confounders, and effect modifiers. Give diagnostic criteria, if applicable This was thoroughly explained in the Methods section, subtitle ‘Measures’, on pages 4, 5 and 6 of the ‘MainDocument’. |
| Data sources/ measurement | 8* | For each variable of interest, give sources of data and details of methods of assessment (measurement). Describe comparability of assessment methods if there is more than one group All necessary information is given in the subsection ‘Measures’ on pages 4, 5 and 6 of the ‘MainDocument’. |
| Bias | 9 | Describe any efforts to address potential sources of bias This was explained in the ‘Data Analyses’ section of the manuscript. |
| Study size | 10 | Explain how the study size was arrived at This was explained in the Methods section (page 4, 5 and 7 of the ‘MainDocument’). |
| Quantitative variables | 11 | Explain how quantitative variables were handled in the analyses. If applicable, describe which groupings were chosen and why See data analyses section, page 7-8 of the ‘MainDocument’. |
| Statistical methods | 12 | (*a*) Describe all statistical methods, including those used to control for confounding |
|  |  | (*b*) Describe any methods used to examine subgroups and interactions |
|  |  | (*c*) Explain how missing data were addressed |
|  |  | (*d*) If applicable, describe analytical methods taking account of sampling strategy |
|  |  | (*e*) Describe any sensitivity analyses  A, b, c, d and e are all thoroughly explained in the Data analyses section, pages 7-8 of the ‘MainDocument’. |
| Results | | |
| Participants | 13* | (a) Report numbers of individuals at each stage of study—eg numbers potentially eligible, examined for eligibility, confirmed eligible, included in the study, completing follow-up, and analysed  These numbers are provided on page 4, 5 and 7 of the ‘MainDocument’ |
|  |  | (b) Give reasons for non-participation at each stage see pages 4,5 and 7 of the ‘MainDocument’ |
|  |  | (c) Consider use of a flow diagram We did not add a flow diagram because the ‘flow’ of participants is not very complex in this study. We are convinced that the explanation in the text is sufficiently clear. |
| Descriptive data | 14* | (a) Give characteristics of study participants (eg demographic, clinical, social) and information on exposures and potential confounders See Table 1 and Results section on page 9 of the ‘MainDocument’. Information regarding confounders is also provided in the Methods section, page 6 of the ‘MainDocument’. |
|  |  | (b) Indicate number of participants with missing data for each variable of interest This was reported in Table 1 |
| Outcome data | 15* | Report numbers of outcome events or summary measures See Table 1 and text on page 9 of the MainDocument |
| Main results | 16 | (*a*) Give unadjusted estimates and, if applicable, confounder-adjusted estimates and their precision (eg, 95% confidence interval). Make clear which confounders were adjusted for and why they were included See Tables 2-5 and Data analyses section (page 7 and 8 of the ‘MainDocument’). |
|  |  | (*b*) Report category boundaries when continuous variables were categorized See Methods section (Measures) |
|  |  | (*c*) If relevant, consider translating estimates of relative risk into absolute risk for a meaningful time period This is not relevant for our manuscript |
| Other analyses | 17 | Report other analyses done—eg analyses of subgroups and interactions, and sensitivity analyses Results of sensitivity analyses are presented in the Supplementary files, results of the interactions (second aim) are presented in Tables 4 and 5 |
| Discussion | | |
| Key results | 18 | Summarise key results with reference to study objectives The key results were summarized in the first paragraph of the Discussion, see page 11 of the ‘MainDocument’ |
| Limitations | 19 | Discuss limitations of the study, taking into account sources of potential bias or imprecision. Discuss both direction and magnitude of any potential bias Limitations of the study were discussed on page 14 of the ‘MainDocument’. |
| Interpretation | 20 | Give a cautious overall interpretation of results considering objectives, limitations, multiplicity of analyses, results from similar studies, and other relevant evidence This was done in the Discussion section of the paper (pages 11 to 15 in the ‘MainDocument’. |
| Generalisability | 21 | Discuss the generalisability (external validity) of the study results Because of the multi-country approach, generalisability is high, but because of small samples in specific countries, generalisability is rather limited. These nuances are discussed in the Strengths and Limitations section (page 14) of the ‘MainDocument’. |
| Other information | | |
| Funding | 22 | Give the source of funding and the role of the funders for the present study and, if applicable, for the original study on which the present article is based Funding information is given on the Title Page |

*Give information separately for exposed and unexposed groups.

**Note:** An Explanation and Elaboration article discusses each checklist item and gives methodological background and published examples of transparent reporting. The STROBE checklist is best used in conjunction with this article (freely available on the Web sites of PLoS Medicine at http://www.plosmedicine.org/, Annals of Internal Medicine at http://www.annals.org/, and Epidemiology at http://www.epidem.com/). Information on the STROBE Initiative is available at www.strobe-statement.org.
